# MesoTRAP: a feasibility study that includes a pilot clinical trial comparing video-assisted thoracoscopic partial pleurectomy decortication with indwelling pleural catheter in patients with trapped lung due to malignant pleural mesothelioma designed to address recruitment and randomisation uncertainties and sample size requirements for a phase III trial

**DOI:** 10.1136/bmjresp-2018-000368

**Published:** 2019-01-05

**Authors:** Claire Matthews, Carol Freeman, Linda D Sharples, Julia Fox-Rushby, Angela Tod, Nicholas A Maskell, John G Edwards, Aman S Coonar, Pasupathy Sivasothy, Victoria Hughes, Najib M Rahman, David A Waller, Robert Campbell Rintoul

**Affiliations:** 1 Papworth Trials Unit Collaboration, Royal Papworth Hospital NHS Foundation Trust, Cambridge, UK; 2 Department of Medical Statistics, London School of Hygiene & Tropical Medicine, London, UK; 3 Department of Primary Care and Public Health Sciences, King's College London, London, UK; 4 School of Nursing and Midwifery, University of Sheffield, Sheffield, UK; 5 Academic Respiratory Unit, University of Bristol, Bristol, UK; 6 Department of Thoracic Surgery, Sheffield Teaching Hospitals NHS Foundation Trust, Sheffield, UK; 7 Department of Thoracic Surgery, Royal Papworth Hospital NHS Foundation Trust, Cambridge, UK; 8 Department of Medicine, Cambridge University Hospitals, Cambridge, UK; 9 Nuffield Department of Medicine, University of Oxford, Oxford, UK; 10 Department of Thoracic Surgery, St Bartholomew’s Hospital, London, UK; 11 Department of Oncology, University of Cambridge, Cambridge, UK

**Keywords:** mesothelioma, pleural disease, thoracic surgery, asbestos induced lung disease

## Abstract

**Introduction:**

One of the most debilitating symptoms of malignant pleural mesothelioma (MPM) is dyspnoea caused by pleural effusion. MPM can be complicated by the presence of tumour on the visceral pleura preventing the lung from re-expanding, known as trapped lung (TL). There is currently no consensus on the best way to manage TL. One approach is insertion of an indwelling pleural catheter (IPC) under local anaesthesia. Another is video-assisted thoracoscopic partial pleurectomy/decortication (VAT-PD). Performed under general anaesthesia, VAT-PD permits surgical removal of the rind of tumour from the visceral pleura thereby allowing the lung to fully re-expand.

**Methods and analysis:**

MesoTRAP is a feasibility study that includes a pilot multicentre, randomised controlled clinical trial comparing VAT-PD with IPC in patients with TL and pleural effusion due to MPM. The primary objective is to measure the SD of visual analogue scale scores for dyspnoea following randomisation and examine the patterns of change over time in each treatment group. Secondary objectives include documenting survival and adverse events, estimating the incidence and prevalence of TL in patients with MPM, examining completion of alternative forms of data capture for economic evaluation and determining the ability to randomise 38 patients in 18 months.

**Ethics and dissemination:**

This study was approved by the East of England-Cambridge Central Research Ethics Committee and the Health Research Authority (reference number 16/EE/0370). We aim to publish the outputs of this work in international peer-reviewed journals compliant with an Open Access policy.

**Trial registration:**

NCT03412357.

## Introduction

Malignant pleural mesothelioma (MPM) is a rare cancer affecting the pleura, closely associated with previous exposure to asbestos. Although the import and use of asbestos has been banned in over 60 countries worldwide, including the UK, MPM remains a major clinical and public health problem.^1^

MPM caused 2542 deaths in the UK in 2015, and globally, mesothelioma is estimated to cause 38 400 deaths per year.^2 3^ Epidemiological data indicate that 65 000 deaths are expected between 2002 and 2050 in the UK alone.^4^ Currently, median survival is around 9.5 months, there is no known cure and treatment is palliative.^5^ Only Pemetrexed and platinum-based chemotherapy have been shown to offer a significant benefit providing a modest survival increase of 8–10 weeks.^6^

One of the most debilitating symptoms for patients with MPM is breathlessness caused by the build-up of fluid in the pleural space. As the pleural effusion increases, symptoms become more severe, and patients are often referred for drainage and a talc pleurodesis, with a view to preventing recurrence.

An effective pleurodesis is dependent on apposition of visceral and parietal pleura. In mesothelioma, it is common for tumour to be present on the visceral surface of the lung. This can prevent the lung from fully reinflating following fluid removal, meaning that the visceral and parietal pleura cannot appose. This situation is called ‘trapped lung’ (TL).

When the lung is trapped, the fluid recurs leading to repetitive cycles of breathlessness, drainage and fluid reaccumulation. This leads to repeated hospital attendances with associated healthcare costs as well as distress and inconvenience to the patient and their families. Furthermore, the pleural space can become loculated such that subsequent aspirations/drains are less effective, and the risk of pleural infection is increased with subsequent morbidity and mortality.

Rather than repeated pleural aspirations/drains, some clinicians are now using an indwelling pleural catheter (IPC) to manage TL. Inserted under local anaesthesia as a day case procedure, an IPC is a soft silicone catheter with a one-way valve at the distal end. Generally well tolerated, it can drain fluid for weeks to months. Spontaneous pleurodesis is thought to develop in around 20% of cases, allowing the IPC to be removed.^7 8^ However, complications such as pleural infection (<5%), blockage (20%) or displacement can occur requiring removal or replacement, and for some, the presence of the catheter acts as a constant reminder of the underlying disease.^9 10^ Placement of an IPC may also be dependent on availability of a community-based healthcare professional to drain fluid 2–3 times weekly if the patient cannot manage this independently.

An alternative approach often favoured by thoracic surgeons is video-assisted thoracoscopic partial pleurectomy/decortication (VAT-PD).^11^ Performed under general anaesthesia, VAT-PD permits surgical removal of the rind of tumour from the visceral pleura, thereby allowing the lung to fully expand again. Simultaneous removal of mesothelioma from the parietal pleura allows pleurodesis to occur. The advantage of this approach is that TL and pleurodesis are treated in one procedure, but disadvantages include the requirement for general anaesthesia, an inpatient stay of up to 7 days and a postoperative serious adverse event (SAE) rate of 17%.^12^

We searched MEDLINE, Embase, Cumulative Index to Nursing and Allied Health Literature and the Cochrane Library for articles about the management of TL by IPC and VAT-PD in patients with mesothelioma using the keywords ‘mesothelioma’, ‘entrap* lung*’, ‘encase* lung*’ or ‘restrictive pleuris*‘ or ‘unexpand* lung*'.

The prevalence of TL in MPM is poorly documented. In case series of malignant pleural effusion with TL, the underlying aetiology was MPM in 13%–37% of cases.^13–15^

No randomised trials comparing IPC with VAT-PD for management of TL in MPM (or other lung malignancy) have been reported. However, several small, retrospective mixed tumour type series reporting IPC use in TL indicate that IPCs may be safe, reasonably effective at controlling dyspnoea and that their use could reduce repeated admissions to hospital.^16–18^

With regard to VAT-PD, there are no published studies specifically addressing the management of TL in MPM. However, the 2011 European Respiratory Society/European Society of Thoracic Surgery (ERS/ESTS) MPM guidelines recommended that pleurectomy/decortication is considered for symptomatic patients with TL (recommendation grade 2C) and that a VATS approach is preferred (grade 1C).^19^ There is also little research examining the understanding of surgical treatments for MPM or exploring factors influencing willingness to participate in MPM trials or decisions regarding randomisation.

The MesoVATS trial randomised patients with MPM to talc pleurodesis versus VAT-partial pleurectomy.^12^ Although there was no difference in median survival between the two arms, there was some evidence that surviving patients in the VAT arm had better quality of life from 6 months post-treatment than surviving patients in the talc pleurodesis arm. However, because of the inclusion criteria, there were very few cases of TL in MesoVATS, and therefore, the outcomes are not directly applicable to MPM with TL and pleural effusion. Searches on ClinicalTrials.gov show there are no ongoing studies examining IPC versus VAT-PD for TL in MPM.

The rationale for undertaking the MesoTRAP study is to begin to provide high-quality evidence for the best management of TL, which affects a significant percentage of patients with MPM in their final months of life. TL is a challenging condition to manage and is associated with high morbidity; therefore, a study investigating the two most commonly used approaches, namely IPC and VAT-PD, is timely.

The objectives of this study are to determine whether it is possible to identify, recruit and randomise patients to a trial of insertion of an IPC versus VAT-PD in TL due to MPM and then, following randomisation, to measure dyspnoea and chest pain using visual analogue scale (VAS) scores, assess post-treatment complications, measure resource and health service use and monitor quality of life in each treatment group.

If the pilot clinical trial is successful in recruiting and randomising 38 patients in the 18-month timeline and there is no evidence of patient harm from study interventions (when comparing one randomised group with the other), we plan to develop the trial into a full phase III study to compare the efficacy of IPC versus VAT-PD for managing TL with pleural effusion in MPM.

There has been one Substantial Amendment to the study protocol to date as described below:

Pilot clinical trial:

1. Removal of the exclusion criterion ‘IPC in situ for more than 28 days’.
2. Removal of the exclusion criterion ‘Previous attempt at pleurodesis on ipsilateral side’.
3. Change in wording of inclusion criterion from ‘Confirmed MPM’ to ‘Pathologically confirmed MPM’.

Overall feasibility study

1. Observational sub-study added

## Methods and analysis

### Study design

This is a feasibility study that includes a pilot multicentre, open-label randomised controlled clinical trial designed to measure the SD, and examine the patterns of change over time, of VAS scores for dyspnoea following randomisation in each treatment group.

Secondary objectives of the feasibility study include estimation of the following:

- SD of VAS scores for chest pain.
- Quality of life at baseline, intervention, 6 weeks, 3, 6 and 12 months postrandomisation.
- Survival and adverse events.
- The prevalence of TL in patients with MPM.
- The percentage of eligible patients in participating centres.
- The ability to recruit and randomise 38 patients to either VAT-PD versus IPC within 18 months in patients with TL and pleural effusion due to MPM.
- Completion rates for alternative forms of data capture for health service and resource use data for economic evaluation.

The full protocol is available as online supplementary file 1.

10.1136/bmjresp-2018-000368.supp1 Supplementary data

## Pilot clinical trial

### Inclusion and exclusion criteria

The primary inclusion criterion is the presence of TL defined as ‘clinically significant TL requiring intervention in the opinion of the clinical team’ in a patient with proven MPM. A pleural effusion must be present and the patient must be ≥18 years old, able to give informed consent and be considered by the clinical team to be expected to survive at least 4 months as well as being suitable for, and willing to undergo, treatment with either VAT-PD or IPC.

The main exclusion criteria are full lung re-expansion following pleural drainage and evidence of active pleural infection.

### Patient groups

It is anticipated that eligible patients will come from one of two groups:

- Group 1: patients found to have TL following fluid drainage by aspiration/intercostal chest drain or post-thoracoscopy.
- Group 2: Patients found to have TL following placement of IPC for management of pleural effusion. Patients in this group will be eligible to be recruited and randomised to either VAT-PD or continuation with the IPC as long as all other inclusion/exclusion criteria are met.

The study will be undertaken at mesothelioma surgical centres across the UK with expertise in both IPC and VAT-PD together with their linked non-surgical referral hospitals. Patients from non-surgical centres who are randomised to VAT-PD will be referred to their nearest surgical centre for the procedure to be carried out.

### Participant identification and informed consent

**Figure 1:**
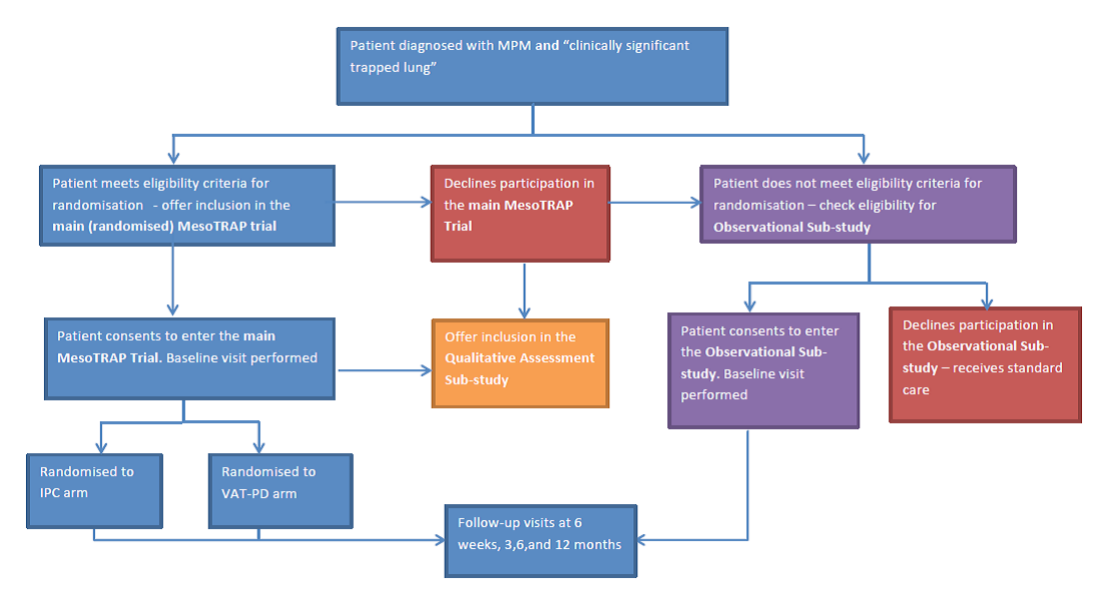
Study flow chart. IPC, indwelling pleural catheter; MPM, malignant pleural mesothelioma; VAT-PD, video-assisted thoracoscopic partial pleurectomy decortication. MesoTRAP Investigators (6 August 2018).

Patients meeting the eligibility criteria will be identified and approached by the research team at their local centre (figure 1). Patients will be provided with a patient information sheet (PIS) and be given a minimum of 24 hours to consider participation. Informed consent will be taken by one of the study doctors or research nurses (see online supplementary file 2).

10.1136/bmjresp-2018-000368.supp2 Supplementary data

A list of current participating sites is provided as online supplementary file 3.

10.1136/bmjresp-2018-000368.supp3 Supplementary data

### Randomisation

Following provision of consent, baseline measurements will be taken, suitable patients will be randomised and their procedure date will be arranged.

Patients will be randomised in a 1:1 ratio using a computer-generated minimisation program with a random element such that each patient retains a non-zero probability of being randomised to each of the treatment arms and groups are well-balanced, with minimisation factors:

- High risk as defined by European Organisation for Research and Treatment of Cancer (EORTC) mesothelioma risk score (high/low risk).^20^ Patients will be defined as high risk if they meet three or more of:
  - White cell count >8.3×10^9^/L, tested on the day of randomisation or within previous 7 days.
  - Non-epithelioid type (unknown type is classed as non-epithelioid).
  - Male.
  - Eastern Cooperative Oncology Group performance status of ≥1.
- Previous insertion of IPC on same side as effusion requiring management.

Due to the nature of the interventions, there will be no blinding of the treatment allocations.

### Sample size

The sample size is chosen to be feasible within the timescale of the study in order to estimate the prevalence of TL, feasibility of recruitment and randomisation and estimate the SD and the patterns of change over time of the VAS measurements. Browne^21^ provides justification of sample sizes in pilot studies and shows that a sample of 38 is appropriate (allowing for a 20% failure to record any dyspnoea), provided that any subsequent definitive trial is based on the 70%–80% upper confidence limit for the SD rather than the sample estimate itself. Although Teare *et al* 22recommends larger sample sizes on the basis that, over all trial phases, they are more efficient than inflating the SD based on confidence limits, a much larger study is not feasible within a reasonable time frame. Therefore, we take the approach of Browne and a sample of 38 patients.

### Study interventions

Unlike many clinical trials in which an investigational arm is compared against a standard practice arm, MesoTRAP is different in that there is no accepted standard treatment for TL in mesothelioma at present. MesoTRAP has been designed to begin a comparison of these two options in terms of patient benefit. Patients will only be randomised if their clinician is confident that they are suitable for both IPC and VAT-PD. At present, we do not know what the difference in patient benefit is between the two interventions.

Investigators at all sites agree to adhere to study-specific standard operating procedures for performing VAT-PD and IPC procedures.

#### Video-assisted thoracoscopic partial pleurectomy/decortication

Under general anaesthesia, a thoracic surgeon creates an initial port in the chest wall and the pleural effusion is drained to dryness. Additional ports are optional to achieve lung expansion. Thoracotomy (with rib spreading) is prohibited. Sharp and blunt dissection of the visceral pleura is mandatory to release TL. A parietal pleurectomy is optional by developing an extrapleural plane. This dissection plane is extended as widely as possible. Resection of the diaphragmatic and pericardial pleura are optional but not generally performed. At the end of the procedure, one or more intercostal drains are placed, with the use of suction optional. The median length of stay for VAT-PD was 7 days (IQR 5–11 days) in MesoVATS.^12^

#### Indwelling pleural catheter

Inserted under local anaesthesia as a day case, a soft silicone IPC with a one-way valve at the distal end is tunnelled a few centimetres under the skin. The proximal part is inserted into the pleural space, and the distal valve is connected to a vacuum drainage bottle. The IPC can be drained at home by the patient, carer or district nurse as often as required.

### Criteria for modifying or discontinuing allocated intervention

If a patient, randomised to VAT-PD deteriorates to the point that they are not fit enough to undergo VAT-PD, they will be offered an IPC instead. This decision will be at the discretion of the clinical team managing the patient and will be recorded and reported. For patients randomised to the IPC arm, the IPC may be removed if there is no significant drainage for 4 weeks and no radiological evidence of significant fluid reaccumulation. All recruited patients will be reported.

### Data collection

Detailed screening logs will be collected from sites on a monthly basis, and these will be used to assess the prevalence of TL.

Patients will be followed up according to the visit schedule (table 1). Clinical and health resource use data will be collected at baseline, and the patient will be asked to complete two quality of life questionnaires (EQ-5D-5L and EORTC QLQC30) along with VAS scores for chest pain and dyspnoea. Patients will be given paper diaries to record their VAS score for chest pain and dyspnoea daily for 6 weeks, then weekly up to 12 months. Measurements of the VAS scores will be taken according to an agreed standard operating procedure with duplicate measurements being taken at the coordinating centre. Patients who are randomised to IPC will be given a paper diary to record their fluid drainage from randomisation to study completion.

**Table 1:** Schedule of events

Specific activity	Screening	Baseline/ randomisation	Intervention (0–3 weeks postrandomisation)	6 weeks±1 week	3 months±1 week	6 months±1 weeks	12 months±1 weeks
Check eligibility of potential participant	**X**						
Provide patient information sheet	**X**						
Take informed consent		**X**					
Baseline clinical data collection		**X**					
Randomisation		**X**					
VAT-PD or IPC			**X**				
VAS scores for dyspnoea and chest pain		**X**	**X**	**X**	**X**	**X**	**X**
EQ-5D and EORTC QLQC30		**X**	**X**	**X**	**X**	**X**	**X**
Review/reporting of patient AEs/SAEs		**X**	**X**	**X**	**X**	**X**	**X**
Qualitative interviews				**X**			
Clinical follow-up data				**X**	**X**	**X**	**X**
Health service and resource use data			**X**	**X**	**X**	**X**	**X**

AEs, adverse events; EORTC, European Organisation for Research and Treatment of Cancer Quality Life Questionnaire; EQ, EuroQoL; IPC, indwelling pleural catheter; SAEs, serious adverse events; VAS, visual analogue scale; VAT-PD, video-assisted thoracoscopic partial pleurectomy/decortication.

Clinical data, adverse events, health resource use data and quality of life questionnaires will be completed at the time of the study interventions and at each follow-up visit. Follow-up visits at 6 weeks, 3, 6 and 12 months postrandomisation are planned to coincide with standard clinical care visits.

All data will be entered remotely onto a secure bespoke database.

### Adverse events

All SAEs occurring between randomisation and the end of follow-up will be recorded in the hospital notes and case report forms. Unexpected SAEs will be submitted to the sponsor within 24 hours of the site becoming aware. The sponsor will report any unexpected SAEs to the Data Monitoring Committee (DMC). If an SAE occurs that is considered to be both unexpected and related to the study protocol (SUSAR), it will be reported within 24 hours of recognition. The sponsor will report any Suspected Unexpected Serious Adverse Reactions (SUSARs) to the research ethics committee within 15 days of their knowledge of the event, and local Principal Investigators (PIs) will be notified.

Non-SAEs will be not be recorded or reported unless they form part of the clinical event dataset.

### Statistical analysis

#### Primary analysis

The SD for the VAS dyspnoea score to be used in a phase III trial will be estimated as the 70% upper limit of the CI as recommended in Browne.^21^ The patterns of change over time will be assessed using descriptive statistics and graphical representations.

Our primary analysis will use the intention to treat, since this will give a more reproducible estimate of the treatment effect and its SD, although we will investigate any disagreement between this and the per protocol estimate.

#### Secondary analyses

- The SD for the VAS chest pain score to be used in a phase III trial will be estimated as the 70% upper limit of the CI as recommended in Browne.^21^ The patterns of change over time will be assessed using descriptive statistics and graphical representations.
- EQ-5D-5L and EORTC QLQ-C30 scores will be summarised by treatment group via descriptive statistics in order to examine quality of life post-intervention. Scores will also be summarised descriptively and graphically over time in order to assess patterns of change. Questionnaire completion rates and data quality will be examined.
- The survival rate at 30 days and 12 months postrandomisation will be summarised by treatment arm.
- SAEs will be recorded from randomisation until the end of the follow-up period and will be reported by treatment group.
- The recruitment rate will be estimated as the number of patients with TL due to MPM recruited, divided by the number of respective patients identified as eligible; it will be expressed as a rate per centre, per month open for recruitment.
- The prevalence of TL in patients with MPM will be estimated as the number of MPM patients with pleural effusion and TL divided by the number of MPM patients within the 18-month study period. This will be multiplied by 100 and reported as a percentage.
- The percentage of eligible patients in participating centres will be estimated as the number of eligible patients divided by the number of MPM patients with TL screened in each centre, multiplied by 100.

A detailed statistical analysis plan will be finalised before the end of recruitment. Briefly, the repeated assessments of continuous measurements (postrandomisation VAS scores for dyspnoea and pain and quality of life indices) will be analysed using linear mixed models, exploring linear and higher polynomial relationships between outcomes and time after randomisation. Patients will be included as random effects on the intercept and, if appropriate, the coefficients. The baseline measurements, treatment arm, (if possible) minimisation factors and time since randomisation will be included as fixed effects. A treatment by time interaction will also be investigated. Models will be fitted using restricted maximum likelihood, and all point estimates and components of variation will be retrieved. Model assessment will be by examination of residuals and influence statistics.

Mean differences between outcomes, overall and restricted to the first year after randomisation will be taken as the primary measures of treatment effect. Patients who die will be assigned the worst score possible (100 for dyspnoea and pain) and the area under the VAS curve for each patient, estimated from the above models, will be calculated. The variance of these outcomes will be estimated from the models and used in the sample size estimates for a phase III trial.

No subgroup analyses are planned.

### Trial oversight

The Trial Management Group, responsible for the day-to-day running of the overall feasibility study, will meet at least every 2 months to discuss recruitment, safety, data management and local site issues.

The Trial Steering Committee will meet 6 monthly (or more frequently if necessary) to monitor and supervise the trial, to ensure it is being conducted according to the protocol and timelines, to review any relevant information from other sources (eg, other related trials) and to consider recommendations from the DMC.

Annual DMC meetings will review progress against the agreed milestones, recruitment and safety. The committee will consist of experienced, independent personnel. The DMC will meet after the first 15 patients are randomised to review the data for safety. Meetings will be held as necessary should urgent issues arise.

Data monitoring will be conducted remotely by the trial manager and data manager. Site visits will be conducted if triggered, for example, by safety concerns or suspected protocol non-compliance.

## Health economic feasibility study

During the trial, the economic study will evaluate alternative data collection mechanisms, with a view to informing future trial design. It will therefore:

1. Design bespoke data collection forms for interventions and their follow-up, with inputs from individual study centres.
2. Evaluate the suitability of collecting follow-up health services use data from patients and via routine data sources.
3. Develop a data collection and analysis plan for a future trial.

## Observational substudy

In parallel with the main study, an observational substudy will collect observational data on a cohort of patients who have MPM and TL but who are either not eligible to participate, or who decline to participate in the main study. Patients in the observational substudy will receive the same baseline and follow-up visits as those in the main study but will receive standard clinical care.

The main inclusion criterial are pathologically confirmed MPM and the presence of TL, defined as a ‘clinically significant TL in the opinion of the clinical team’. Patients must also be ≥18 years old and able to give informed consent. The only exclusion criterion is the lung re-expanding fully following pleural fluid drainage, that is, no entrapment.

Patients meeting the eligibility criteria will be informed about the study, provided with a PIS and given at least 1 hour to consider participation. A member of the research team will address any questions and take written informed consent (see online supplementary file 4).

10.1136/bmjresp-2018-000368.supp4 Supplementary data

Following provision of consent the baseline visit will be conducted, with follow-up visits at 6 weeks, 3, 6 and 12 months postbaseline. Patient follow-up and data collection will match that used in the pilot clinical trial.

## Qualitative substudy

A qualitative substudy will also examine patient experience of the interventions and factors influencing patient decisions to participate and accept randomisation or not. Five patients randomised to the VAT-PD group, five patients randomised to the IPC group and five who decline participation will be recruited to participate in a semistructured interview. Informed consent will take place at the patient’s local participating centre (see online supplementary file 5), and patient will undergo a telephone interview with a specialist nurse from the coordinating centre. Framework analysis methods will be used.^23^ The interviews will explore the following questions:

10.1136/bmjresp-2018-000368.supp5 Supplementary data

1. What is the patient experience of the MesoTRAP recruitment process?
2. What factors influence patient decisions regarding MesoTRAP including participation and randomisation?
3. What is the patient experience of MesoTRAP study interventions?
4. What are the implications of the findings for MesoTRAP if it moves to a full study in terms of design, patient information and support?

## Ethics and dissemination

We aim to publish the outputs of this work in an international peer reviewed journal compliant with an Open Access policy. The work will be submitted to major national and international clinical meetings and we will inform patient with mesothelioma/carer support groups of the results including Mesothelioma UK, Clydeside Action on Asbestos, Mick Knighton Mesothelioma Research Fund and the Greater Manchester Asbestos Victims Support Group, a number of whom produce newsletters for their members/supporters.

**Table 2:** MesoTRAP investigators (6 August 2018)

Mr Kelvin Lau	St Bartholomew's Hospital, London
Mr M Nidal Bittar	Victoria Hospital, Blackpool
Mr Antonio Martin-Ucar	University Hospitals, Coventry
Dr Jurgen Herre	Cambridge University Hospitals
Dr Paul Beckett	Royal Derby Hospital
Mr Alan Kirk	Golden Jubilee National Hospital, Scotland
Dr Kevin Blyth	Queen Elizabeth University Hospital, Glasgow
Mr Apostolos Nakas	Glenfield Hospital, Leicester
Dr Eleanor Mishra	Norfolk and Norwich University Hospitals
Dr Shahul Khan	Royal Stoke University Hospital
Dr Helen Roberts	Nottingham University Hospitals NHS Trust
Mr Dionisios Stavroulis	John Radcliffe Hospital, Oxford
Dr Louise Brown	The Pennine Acute Hospitals NHS Trust
Dr Mohammed Munawar	LancashireTeaching Hospitals
Dr Matthew Evison	University Hospital of South Manchester
