# Supplementary material for: MesoTRAP: a feasibility study that includes a pilot clinical trial comparing video-assisted thoracoscopic partial pleurectomy decortication with indwelling pleural catheter in patients with trapped lung due to malignant pleural mesothelioma designed to address recruitment and randomisation uncertainties and sample size requirements for a phase III trial
Source: BMJ Open Respir Res. 2019 Jan 5;6(1):e000368. doi: 10.1136/bmjresp-2018-000368 (PMC6326291; doi:10.1136/bmjresp-2018-000368)
Supplement: Supplementary data [file bmjresp-2018-000368supp001.pdf]

Papworth Hospital NHS
NHS Foundation Trust

MesoTRAP Clinical Study Protocol

**Study Title**	**MesoTRAP:** A pilot clinical trial and feasibility study comparing video-assisted thoracoscopic partial pleurectomy/decortication with indwelling pleural catheter in patients with trapped lung due to malignant pleural mesothelioma designed to address recruitment and randomisation uncertainties and sample size requirements for a Phase III trial.	
**Chief investigator**	**Dr Robert Rintoul PhD FRCP**<br>Director, Papworth Trials Unit Collaboration<br>Consultant Respiratory Physician, Lead Clinician for Cancer, Papworth Hospital NHS foundation Trust<br>Department of Thoracic Oncology, Papworth Hospital, Cambridge CB23 3RE;<br>Tel: 01480 364342 Fax: 01480 364331, Email: robert.rintoul@nhs.net	
**Trial Sponsor**	**Papworth Hospital NHS Foundation Trust**<br>Research & Development Unit, Papworth Hospital, Cambridge CB23 3RE<br>Tel: 01480 364143 Fax: 01480 364550, Email: R&DAdmin@papworth.nhs.uk	
**Trial Funder**	**NIHR Research for Patient Benefit Committee**<br>RfPB reference: PB-PG-1014-35050	
**Trial Management group**	Professor Linda Sharples	London School of Hygiene and Tropical Medicine
	Professor Julia Fox-Rushby	King's College London
	Dr Nick Maskell	North Bristol NHS Trust
	Dr Najib Rahman	Oxford University Hospitals Trust
	Mr Aman Coonar	Papworth Hospital NHS Foundation Trust
	Ms Carol Freeman	Papworth Trials Unit Collaboration
	Dr Vikki Hughes	Papworth Trials Unit Collaboration
	Mr John Edwards	Sheffield Teaching Hospitals NHS Trust
	Mr David Waller	Barts Health NHS Trust
	Professor Angela Mary Tod	University of Sheffield
	Ms Claire Matthews	Papworth Hospital NHS Foundation Trust
	Mr Tom Devine	Papworth Hospital NHS Foundation Trust
	Ms Kate Slaven	Papworth Hospital NHS Foundation Trust
**Study location**	UK	Multicentre

Papworth Hospital NHS
NHS Foundation Trust

1 STUDY SYNOPSIS

**Title**	**MesoTRAP:** A pilot clinical trial and feasibility study comparing video-assisted thoracoscopic partial pleurectomy/decortication with indwelling pleural catheter in patients with trapped lung due to malignant pleural mesothelioma designed to address recruitment and randomisation uncertainties and sample size requirements for a Phase III trial.
**Sponsor**	Papworth Hospital NHS Foundation Trust
**Medical condition**	Malignant Pleural Mesothelioma with Trapped Lung
**Purpose**	The overarching aim of our research programme is to determine the best treatment for managing trapped lung in patients with malignant pleural mesothelioma (MPM) and pleural effusion. Trapped lung (TL) is a cause of significant morbidity in the final months of life causing dyspnoea (breathlessness), chest pain and repeated medical procedures to drain recurrent pleural fluid necessitating multiple hospital visits/in-patient days.<br><br>We plan to undertake a full Phase III randomised controlled trial of video-assisted thoracoscopic partial pleurectomy/decortication (VAT-PD) versus indwelling pleural catheter (IPC) to determine the best method of controlling/palliating dyspnoea and chest pain, the principal symptoms in MPM with trapped lung and pleural effusion.<br><br>However, we recognise that prior to undertaking a full study, there are some uncertainties that need to be addressed to inform the best design for a Phase III study. These are:<br><br>i) What are the standard deviations of Visual Analogue Scale scores for dyspnoea in each treatment group following randomisation?<br><br>ii) Will patients accept randomisation to IPC or VAT-PD in a real life trial situation?<br><br>iii) How prevalent is trapped lung in MPM?<br><br>We will also investigate the feasibility of data collection formats for a future cost-effectiveness analysis.
**Primary objective**	The primary objective of this pilot clinical trial and feasibility study is to measure the standard deviation of Visual Analogue Scale scores for dyspnoea following randomisation and examine the patterns of change over time in each treatment group.

Papworth Hospital NHS
NHS Foundation Trust

**Secondary objectives**	To help inform the design of a full Phase III randomized controlled trial we will also aim:<br><br>i) To estimate the standard deviation of Visual Analogue Scale (VAS) scores for chest pain and examine the patterns of change over time in each treatment group.<br>ii) To examine Quality of Life at baseline, intervention, 6 weeks, 3, 6 and 12 months post-randomisation.<br>iii) To document Survival and Adverse Events.<br>iv) To estimate the prevalence of trapped lung in patients with MPM.<br>v) To estimate the percentage of eligible patients in participating centres.<br>vi) To determine the ability to recruit and randomise 38 patients in 18 months into a trial of VAT-PD versus IPC in patients with trapped lung and pleural effusion due to MPM.<br>vii) To investigate the comparative feasibility of alternative forms of data collection for health service and resource use for economic evaluation.
**Trial design**	Multi-centre, open-label, randomised controlled pilot clinical trial and feasibility study.
**Study Endpoints**	i. Visual Analogue Scale scores for dyspnoea, standard deviation and patterns of change over time<br>ii. Visual Analogue Scale scores for chest pain, standard deviation and patterns of change over time<br>iii. Quality of Life at baseline, intervention, 6 weeks, 3, 6 and 12 months post randomisation measured using the EQ-5D-5L and EORTC QLQC30<br>iv. Survival probabilities at 30 days and 12 months post randomisation<br>v. Adverse Events<br>vi. The prevalence of trapped lung in patients with MPM<br>vii. Percentage of eligible patients in participating centres<br>viii. Recruitment rate<br>ix. Data availability for health service and resource use from baseline to 12 months post-randomisation<br>x.
**Sample size**	38 patients randomised
**Eligibility criteria**	***Inclusion Criteria:***<br>1. Pathologically confirmed MPM<br>2. Trapped lung, defined as a *"clinically significant trapped lung requiring intervention in the opinion of the clinical team"*<br>3. Pleural effusion present (following re-accumulation)<br>4. Considered by the clinical team to be suitable and fit enough to undergo VAT-PD

Papworth Hospital NHS
NHS Foundation Trust

	5. Considered by the clinical team to be equally suitable for treatment with VAT-PD or IPC, and therefore eligible for treatment allocation by randomisation.<br>6 Patient willing to receive either VAT-PD or IPC and attend the respective designated centre for their treatment<br>7. Community services or patient/carer able to drain IPC at least twice weekly<br>8 Expected survival of at least 4 months, as assessed by managing clinician<br>9. Age ≥ 18 years<br>10. Able to provide informed consent<br><br>***Exclusion Criteria:***<br>1. Lung re-expands fully following pleural fluid drainage i.e. no entrapment<br>2. Evidence of active pleural infection<br>3. Current participation in an RCT or receiving a CTIMP<br>4. Females: pregnant or lactating
**Screening and Enrolment**	Patients with MPM and pleural effusion will attend their regional mesothelioma centre for review. Those with TL meeting all eligibility criteria will be informed about the study, provided with a patient information sheet and given at least 24 hours to consider participation. At their next research visit, a member of the research team will address any questions and take written informed consent.
**Baseline & Randomisation**	Following consent, patients will be randomised, baseline measurements will be taken and a procedure date will be arranged.<br><br>Patients will be randomised and allocated in a 1:1 ratio, to one of two groups:<br>i) IPC<br>ii) VAT-PD.
**Interventions**	Video-assisted thoracoscopic partial pleurectomy/decortication (VAT-PD) versus Indwelling Pleural Catheter (IPC)
**Follow up**	Follow-up visits at 6 weeks, 3, 6 and 12 months post-randomisation are planned to coincide with clinical care visits.
**End of Study**	Study participation will end when the last patient completes the last visit.

Papworth Hospital NHS
NHS Foundation Trust

**Procedures for safe monitoring**	Serious Adverse Event reports will be forwarded to Papworth Trials Unit Collaboration (PTUC). Reports will be made to the Sponsor and the Data Monitoring Committee (DMC).<br><br>Expected adverse events will be collated and summarised by PTUC and reported to the DMC.
**Criteria for modifying or discontinuing allocated intervention**	If a patient, randomised to VAT-PD, deteriorates to the point that they are not fit enough to undergo VAT-PD, they will be offered an IPC instead. For patients randomised to the IPC arm, the IPC may be removed if there is no significant drainage for 4 weeks and no radiological evidence of significant fluid re-accumulation. All recruited patients will be reported.
**Qualitative Assessment Study:**	In parallel with the main study, a qualitative sub-study (5 patients randomised to the VAT-PD group, 5 patients randomised to the IPC group, and 5 who decline participation) will examine patient experience of the interventions and factors influencing patient decisions to participate and accept randomisation or not.<br><br>• What is the patient experience of the MesoTRAP recruitment process?<br>• What factors influence patient decisions regarding MesoTRAP including participation and randomisation?<br>• What is the patient experience of MesoTRAP study interventions?<br><br>What are the implications of the findings for MesoTRAP if it moves to a full study in terms of design, patient information and support?
**Economic Feasibility Study**	During the feasibility study, the economic study will design and evaluate the data collection mechanisms with a view to informing future trial design. It will therefore:<br><br>1. Design bespoke data collection forms for interventions and their follow up, with inputs from individual study centres.<br>2. Evaluate the suitability of collecting follow-up health services use data from patients and via routine data sources.<br>3. Develop a data collection and analysis plan for a future trial.
**Observational Sub-study**	In parallel with the main study an observational sub-study will collect observational data on a cohort of patients who have MPM and trapped lung, but who are either not eligible to participate, or who decline to participate in the main study. Patients in the Observational Sub-study will receive the same baseline and follow-up visits as those in the main study, but will receive standard clinical care.<br><br>***Inclusion Criteria:***<br>1. Pathologically confirmed MPM<br>2. Trapped lung, defined as a *"clinically significant trapped lung in the opinion of the clinical team"*

	3. Age ≥ 18 years<br>4. Able to provide informed consent<br><br>***Exclusion Criteria:***<br>1. Lung re-expands fully following pleural fluid drainage i.e. no entrapment

Papworth Hospital NHS
NHS Foundation Trust

TABLE OF CONTENTS

1 Study Synopsis 2
2 Introduction 9
2.1 Background 9
2.2 Rationale 10
2.3 Expected Output of Research/Impact 11
3 Trial Objectives 12
3.1 Primary Objective 12
3.2 Secondary Objectives 12
3.3 Study End Points 12
3.3.1 Primary Endpoint 12
3.3.2 Secondary Endpoints 12
4 Trial Design 14
4.1 Statement of design 14
4.2 Study Setting 14
4.3 Sample Size 14
4.4 Diagnosis of trapped lung (TL) 15
5 Participant Recruitment, Randomisation and Follow up 15
5.1 Study Population and eligibility 15
5.2 Patient Groups 15
5.3 Participant identification and informed consent procedure 16
5.4 Randomisation 16
5.5 interventions 17
5.6 Participant follow up 19
6 Data Handling and Record keeping 20
6.1 Data collection, management and analysis 20
6.2 Screening and recruitment LOGS 20
6.3 Baseline and clinical follow up data 21
6.4 Collection of VAS data to inform power calculations for a definitive study. 21
6.5 Quality of Life 22
6.6 Recording and management of Adverse Events 22
6.7 Resource Use Data 22
6.8 Data Monitoring plans 23

Papworth Hospital NHS
NHS Foundation Trust

7 Statistics 23
8 Health Economics 24
9 Qualitative Assessment Sub Study (QASS) 25
9.1 QASS Introduction 25
10 Observational Sub-Study 26
10.1 Observational Sub-Study Introduction 26
10.2 Eligibility 26
10.3 Recruitment and Follow-up 26
11 Project Management 28
11.1 Research Management and governance 28
11.2 Qualitative Sub study 28
11.3 Patient and Public Involvement 28
11.4 Study Registration 28
11.5 Trial Management Group (TMG) 28
11.6 Trial Steering Committee (TSC) 28
11.7 Data Monitoring Committee (DMC) 29
11.8 CRN Eastern 29
12 Ethical & Research Governance approvals 29
12.1 Initial REC and HRA Approval 29
12.2 Site Capability and Capacity 29
12.3 Protocol amendments 30
13 Insurance 30
14 Publication Policy 30
15 References 31
16 Appendix 1: Definitions of Adverse Events 34
16.1 Adverse Event (AE) 34
16.2 Serious Adverse Event 34
16.3 Expected morbidity 34
16.3.1 Expected morbidity following VATS-PD surgery can include: 34
16.3.2 Expected morbidity with an IPC can include: 34

Papworth Hospital NHS
NHS Foundation Trust

2 INTRODUCTION

2.1 BACKGROUND

Malignant pleural mesothelioma (MPM) is a rare type of cancer affecting the pleural lining of the lungs, closely associated with exposure to asbestos. Although more than 20 years have elapsed since a full ban on the import of asbestos into the UK, mesothelioma remains a major clinical and public health problem.

Health and Safety Executive data for 2012 reveals mesothelioma caused 2,535 deaths in the UK (www.hse.gov.uk). Epidemiological data indicates that 65,000 deaths are expected between 2002 and 2050 (Hodgson 2005). Currently, median survival is around 12 months, there is no known cure, and treatment is palliative. Only Pemetrexed and platinum-based chemotherapy has been shown to have a significant benefit providing a modest survival increase of 8-10 weeks (Vogelzang 2003;Bottomley 2006).

One of the most debilitating symptoms for patients with MPM is breathlessness (dyspnoea), caused by the build-up of pleural fluid. As the pleural effusion increases, symptoms become more severe and patients are referred for fluid drainage (via a chest drain) and a talc pleurodesis with a view to prevent fluid from recurring.

An effective pleurodesis is dependent upon apposition of visceral and parietal pleura. In mesothelioma, it is common for tumour to be present on the visceral surface of the lung. This often prevents the lung from fully re-inflating following fluid removal, meaning that the visceral and parietal pleura cannot appose. This situation is called 'entrapped lung' (TL).

When the lung is trapped, the fluid recurs leading to repetitive cycles of breathlessness, fluid drainage and recurrence. This leads to repeated hospital attendances with associated distress and inconvenience to the patient and their families and cost to the NHS. Furthermore, repeated pleural interventions often cause the pleural space to become loculated such that subsequent aspirations/drains are less effective and the risk of pleural infection is increased with subsequent morbidity and mortality.

Rather than repeated pleural aspirations/drains, some clinicians are now using an indwelling pleural catheter (IPC), a soft silicone catheter with a one-way valve at the distal end, inserted under local anaesthesia as a day case procedure to manage TL. Generally well tolerated, they can drain fluid for weeks to months.

Sometimes a natural pleurodesis develops, fluid drainage ceases, and the IPC can be removed. However, complications such as pleural infection (13%), blockage (20%) or displacement can occur requiring removal or replacement, and for some, the presence of the catheter acts as a constant reminder of the underlying disease (Davies 2012).

Placement of an IPC is also dependent upon availability of a community-based health care professional to drain fluid 2-3 times weekly.

An alternative approach often favoured by thoracic surgeons is video-assisted thoracoscopic partial pleurectomy/decortication (VAT-PD) (Rathinam 2013). Performed under general anaesthesia, VAT-PD permits surgical removal of the rind of tumour from the visceral pleura

thereby allowing the lung to fully expand again. Simultaneous removal of mesothelioma from the parietal pleura allows pleurodesis to occur.

The advantage of this approach is that trapped lung and pleurodesis are dealt within one procedure but disadvantages include the requirement for general anaesthesia, an in-patient stay of around 7 days, and a post-operative serious adverse event rate of 17% (Rintoul 2014).

The prevalence of TL in MPM is poorly documented. In case series of malignant pleural effusion with trapped lung, in 13% to 37% of the cases the underlying aetiology was MPM (Bazerbashi 2009; Qureshi 2008; McBrearty 2012). Eligibility data from a pleurodesis trial showed 35% had trapped lung at presentation (personal communication Dr Maskell).

We searched Medline, Embase, Cumulative Index to Nursing and Allied Health Literature and the Cochrane Library for articles about the management of TL by IPC and VAT-PD in patients with mesothelioma using the keywords “mesothelioma”, “entrap* lung*”, “encase* lung*” or “restrictive pleuris*” or “unexpand* lung*”. No randomised trials comparing IPC with VAT-PD for management of TL in MPM (or other lung malignancy) have been reported. There are several small, retrospective mixed tumour type series reporting IPC use in TL (Efthymiou 2009; Kulkarni 2009; Chee 2011). In summary, these showed that IPCs are safe, reasonably effective at controlling dyspnoea and reduce repeated admissions to hospital.

With regard to VAT-PD, there are no published studies specifically addressing the management of TL in MPM. However, the 2011 ERS/ESTS MPM guidelines recommended that pleurectomy/decortication is considered for symptomatic patients with TL (recommendation Grade 2C) and that a VATS approach is preferred (Grade 1C) (Scherpereel 2010). There is also little research examining the understanding of surgical treatments for MPM or exploring factors influencing willingness to participate in MPM trials or decisions regarding randomisation.

The MesoVATS trial, conducted by the applicants and published in 2014, randomised patients with MPM to talc pleurodesis versus VAT-PD (Rintoul et al. 2014). Although there was no difference in median survival between the two arms, there was some evidence that patients in the VAT-PD arm had better quality of life from 6 months post-treatment. However, because of the inclusion criteria, there were very few cases of TL in MesoVATS, and therefore the outcomes are not directly applicable to MPM with TL and pleural effusion. Searches on ClinicalTrials.gov show there are no on-going studies examining IPC versus VAT-PD for TL in MPM.

2.2 RATIONALE

The rationale for undertaking this study is to begin providing high quality evidence for the best management of TL, a scarcely studied and poorly understood condition, which affects a significant percentage of MPM patients in their final months of life. TL is a challenging condition to manage and is associated with high morbidity; therefore a study investigating the two most commonly used approaches, namely IPC and VAT-PD, is timely.

As outlined above, both IPC and VAT-PD have advantages and shortcomings. On one hand, IPC placement is usually straightforward but on-going care is required and there may be late related complications. On the other hand, a VAT-PD is more onerous initially but once a patient recovers, on-going quality of life may be better. Therefore the objectives of this pilot clinical trial and feasibility study are to determine whether it is possible to identify, recruit and randomise

patients to a trial of insertion of an IPC versus VAT-PD in trapped lung due to MPM and then, following randomisation, to measure dyspnoea and chest pain using Visual Analogue Scale scores; assess post treatment complications and monitor quality of life in each treatment group.

2.3 Expected Output of Research/Impact

If the pilot clinical trial and feasibility study is successful in recruiting and randomising 38 patients in the 18 month timeline, and there is no evidence of patient harm from study interventions (when comparing one randomised group with the other), we plan to develop the trial into a full phase III study to compare the efficacy of IPC versus VAT-PD for managing trapped lung with pleural effusion in MPM.

In the full phase III study, the primary (patient-reported) outcome measure will be control of breathlessness using the 100mm VAS dyspnoea score. Data obtained in the pilot clinical trial and feasibility study on the prevalence of trapped lung and the standard deviation of the VAS dyspnoea scores will inform sample size estimates as well as the most appropriate timing for assessing the primary outcome.

Secondary endpoints will include safety; survival; symptoms (chest pain), adverse events, quality of life assessment (EORTC QLQC30 and EQ-5D-5L), and resource/health service use data.

Data collected during the pilot clinical trial and feasibility study on quality of life, safety, and resource/health service use data will also inform the design of a full Phase III study.

Overall, our long-term aim is to identify the best treatment for trapped lung due to MPM in order to improve patient quality of life and morbidity and efficiency of health services. We anticipate that this work (pilot clinical trial and feasibility study and subsequent full study) will inform future NICE guidelines, Cochrane reviews and the management of MPM.

Although this is a UK based study, it will apply to global healthcare. Asbestos is still being used widely in many parts of Eastern Europe and in several developing nations. This means that MPM will be present for many decades to come, even after the worst of the mesothelioma epidemic in the UK and Western Europe is over. Therefore, the lessons learned from MesoTRAP and a subsequent phase III study will be relevant to the care of mesothelioma patients worldwide.

Papworth Hospital NHS
NHS Foundation Trust

3 TRIAL OBJECTIVES

3.1 PRIMARY OBJECTIVE

The primary objective of the pilot clinical trial is to measure the standard deviation of Visual Analogue Scale scores for dyspnoea following randomisation and examine the patterns of change over time in each treatment group.

This will allow estimation of the parameters required to define the primary outcome and sample size of the main Phase III trial.

3.2 SECONDARY OBJECTIVES

To help inform the design of a full Phase III randomized controlled trial we also aim:

1. To estimate the standard deviation of Visual Analogue Scale (VAS) scores for chest pain and examine the patterns of change over time in each treatment group.
2. To examine Quality of Life at baseline, intervention, 6 weeks, 3, 6 and 12 months post-randomisation.
3. To document Survival and Adverse Events.
4. To estimate the prevalence of trapped lung in patients with MPM.
5. To estimate the percentage of eligible patients in participating centres.
6. To determine the ability to recruit and randomise 38 patients in 18 months into a trial of VAT-PD versus IPC in patients with trapped lung and pleural effusion due to MPM.
7. To investigate the comparative feasibility of alternative forms of data collection for health service and resource use for economic evaluation.

3.3 STUDY END POINTS

3.3.1 Primary Endpoint

i. Visual Analogue Scale scores for dyspnoea, their standard deviation and patterns of change over time. The standard deviation for the VAS dyspnoea scale for a subsequent phase III trial will be estimated as the 70% upper limit of the confidence interval as recommended in Browne (1995).

3.3.2 Secondary Endpoints

i. Visual Analogue Scale scores for chest pain, their standard deviation and patterns of change over time. The standard deviation for the VAS chest pain scale for a subsequent phase III trial will be estimated as the 70% upper limit of the confidence interval as recommended in Browne (1995).

ii. Quality of Life will be measured at baseline, at the time of the intervention, at 6 weeks, 3, 6 and 12 months post randomisation using the EQ-5D-5L and EORTC QLQC30 patient-reported QoL questionnaires.

iii. Survival probabilities at 30 days and 12 months post-randomisation

iv. Adverse Events

v. The number of patients with MPM and of patients with TL and pleural effusion due to MPM will be recorded in each centre in order to estimate the prevalence of trapped lung in patients with MPM.

vi. The number of eligible patients with TL and MPM and the number of screened patients with TL and MPM will be recorded in each centre in order to estimate the percentage of eligible patients defined as:

"the number of patients meeting the study inclusion and exclusion criteria in all the participating centres divided by the total number of patients with TL due to MPM in all the participating centres, multiplied by 100".

vii. The recruitment rate will be estimated as the number of patients with TL due to MPM recruited, divided by the number of respective patients identified as eligible and invited to participate and expressed as a rate per centre, per month open for recruitment.

viii. The randomisation rate will be estimated as the number of patients with TL due to MPM who undergo randomisation, divided by the number of respective patients recruited and expressed as a rate per centre, per month open for recruitment.

ix. To compare the completion rates, extent of missing data and accuracy of collecting resource/health service use data during follow-up using patient reports and routine data.

Papworth Hospital NHS
NHS Foundation Trust

4 Trial Design

4.1 Statement of design

This is a multi-centre, open-label, randomised controlled pilot clinical trial and feasibility study comparing video-assisted thoracoscopic partial pleurectomy/decortication (VAT-PD) with indwelling pleural catheter (IPC) in patients with trapped lung (TL) and pleural effusion due to malignant pleural mesothelioma (MPM), aimed at addressing recruitment and randomisation uncertainties as well as sample size requirements for a full phase III study. 38 patients will be randomised and allocated in a 1:1 ratio to either VAT-PD or IPC.

The design of this pilot clinical trial and feasibility study is in line with the IDEAL recommendations for Stage 2b trials undertaken in preparation for a full phase III trial (Pennell 2016). Stage 2b feasibility studies aim to address issues with the design and conduct of a full definitive trial (McCulloch 2009).

4.2 Study Setting

The study will be undertaken at mesothelioma surgical centres with expertise in either IPC, VAT-PD or both procedures, together with their linked non-surgical referral hospitals (hub and spoke). Patients randomised to VAT-PD will attend their nearest surgical centre. Those randomised to IPC will attend their nearest appropriate centre.

4.3 Sample Size

The sample size is chosen to be feasible within the timescale of the study and to allow estimation of the standard deviation and the patterns of change over time of the VAS measurements as well as the prevalence of TL in MPM patients and feasibility of recruitment and randomisation to a full Phase III study.

Browne (1995) provides justification that in pilot studies sample sizes of 30 patients or more are sufficient to estimate a parameter, provided a conservative approach was used to estimate the standard deviation. Therefore, a sample of 38 patient is appropriate (allowing for a 20% failure to record any dyspnoea score, based on previous studies) provided that any subsequent definitive full trial is based on the 70-80% upper confidence limit for the standard deviation of the primary endpoint, rather than the sample estimate itself.

Although Teare (2014) recommends larger sample sizes for feasibility studies on the basis that unadjusted standard deviation estimates from these will result in smaller sample sizes for the definitive trial than if inflated SD estimates (based on confidence limits) are used, a much larger study is not feasible within a reasonable time frame.

Therefore the approach of Browne (1995) is adopted and a sample of 38 patients will be recruited.

4.4 DIAGNOSIS OF TRAPPED LUNG (TL)

No standard definition for TL exists. For the purposes of this study, the diagnosis of TL will be defined as '*clinically significant trapped lung requiring intervention in the opinion of the clinical team'.*

We recognise that there will be considerable variation in what clinicians consider to be 'clinically significant trapped lung' and in order to obtain information to inform future studies we will ask investigators to complete a short questionnaire explaining their rationale and to provide the contemporaneous chest X -ray and CT.

5 PARTICIPANT RECRUITMENT, RANDOMISATION AND FOLLOW UP

5.1 STUDY POPULATION AND ELIGIBILITY

Inclusion Criteria:

1. Pathologically confirmed MPM
2. Trapped lung, defined as a "*clinically significant trapped lung requiring intervention in the opinion of the clinical team"*
3. Pleural effusion present (following re-accumulation)
4. Considered by the clinical team to be suitable and fit enough to undergo VAT-PD
5. Community services or patient/carer able to drain IPC at least twice weekly
6. Considered by the clinical team to be equally suitable for treatment with VAT-PD or IPC, and therefore eligible for treatment allocation by randomisation.
7. Patient willing to receive either VAT-PD or IPC and attend the respective designated centre for their treatment
8. Expected survival of at least 4 months, as assessed by managing clinician
9. Age ≥ 18 years
10. Able to provide informed consent

Exclusion Criteria:

1. Lung re-expands fully following pleural fluid drainage i.e. no entrapment
2. Evidence of active pleural infection
3. Current participation in an RCT or CTIMP
4. Females: pregnant or lactating

5.2 PATIENT GROUPS

It is anticipated that eligible patients will come from one of two groups:

- **Group 1**: Patients found to have TL following fluid drainage by aspiration/intercostal chest drain or post-thoracoscopy.

- **Group 2**: Patients found to have TL following placement of IPC for management of pleural effusion. Patients in this group will be eligible to be recruited and randomised to either VAT-PD or continuation with the IPC as long as all other inclusion/exclusion criteria are met.

5.3 Participant identification and informed consent procedure

Patients with MPM and pleural effusion will attend their regional mesothelioma centre for review.

Those with TL meeting all eligibility criteria will be informed about the study, provided with a patient information sheet and given at least 24 hours to consider participation.

At their next research visit, a member of the research team will address any questions and take written informed consent. Informed consent will include permission to access participants' primary care hospital records through trusts as well as self-reported data from patients.

5.4 Randomisation

Following provision of consent, baseline measurements will be taken, patients will be randomised, and a procedure date will be arranged.

We will give patients 24 hours to read the patient information sheet and consider trial participation. Once a patient has agreed to participate they will be randomised immediately and arrangements will be made to deliver treatment.

In the event that a patient becomes symptomatic due to pleural effusion while awaiting a definitive procedure, fluid will be aspirated as per standard procedure but the participant will go on to receive their randomised treatment. While IPC insertion is likely to be performed within a few days, patients may wait for 2-3 weeks for a VATS-PD procedure (MesoVATS median 14 (IQR 8-21 days). It is recognised that some patients may need fluid drainage while waiting for VATS. Requirement for additional fluid drainage while awaiting surgery will be measured in order to inform future studies.

Randomisation process

Randomisation will be performed by an authorised member of the local research team using an appropriate online system.

Eligibility and consent will be verified, and patients will be randomised and allocated in a 1:1 ratio, to one of two groups:

i) IPC
ii) VAT-PD

Randomisation will be done using a computer-generated minimisation programme incorporating a random element to ensure treatment groups are well-balanced for the following participant characteristics, details of which will also be required for randomisation:

- Stratification using the European Organisation for Research and Treatment of Cancer (EORTC) mesothelioma risk score (high/low risk) *(Curran et al, 1998).* Patients will be defined as high risk if they meet three or more of:

    - white blood cell count >$8.3x10^9$/L, tested on the day of randomisation or within previous 7 days;
    - non-epithelioid type – note that unknown type is classed as non-epithelioid;
    - male;
    - Eastern Cooperative Oncology Group (ECOG) performance status of ⩾1.
- Previous insertion of IPC on same side as effusion requiring management

At randomisation information regarding whether the participant currently has an IPC fitted will also be collected.

Randomisation issues
Randomising between two different treatment modalities (i.e. a ‘medical’ IPC versus a ‘surgical’ VAT-PD) is challenging but can be achieved as it was in MesoVATS, a trial which randomised between Talc Pleurodesis versus VAT-PD (Rintoul 2014).

In MesoVATS only 15.5 % (32/206) of Papworth hospital cases declined randomisation, 10 of the 32 because they did not wish to undergo surgery. However, we feel it is important to assess this issue again given that recruiting patients who already have an IPC *in situ* may render randomisation more challenging. These patients may already be feeling less breathless with their IPC *in situ* and may not wish to be randomised to VAT-PD versus continuation with their IPC.

The assessment of willingness to be randomised, in a real life situation, will be an important part of this pilot clinical trial and feasibility study.

5.5 INTERVENTIONS

Unlike many clinical trials in which an investigational arm is compared against a standard practice arm, MesoTRAP is different in that there is no accepted standard treatment for trapped lung in mesothelioma at present. MesoTRAP has been designed to begin a comparison of these two options in terms of patient benefit. Patients will only be randomised if their clinician is confident that they are suitable for both IPC and VAT-PD. At present we do not know what the difference in patient benefit is between the two interventions.

Video-assisted thoracoscopic partial pleurectomy/decortication (VAT-PD)

Under general anaesthesia a thoracic surgeon creates an initial port in the chest wall and the pleural effusion is drained to dryness. Additional ports may be introduced to achieve lung expansion. Sharp and blunt dissection of the visceral pleura is performed to release trapped lung. A parietal pleurectomy is performed by developing an extrapleural plane. This dissection plane is extended as widely as possible. Generally the diaphragm and pericardium are not incised. At the end of the procedure one or more drains are placed on suction. The median length of stay (LOS) for VAT-PD was 7 days (IQR 5-11 days) in MesoVATS (Rintoul et al., 2014).

Indwelling pleural catheter (IPC)

Inserted under local anaesthesia as a day case, a soft silicone IPC with a one-way valve at the distal end is tunnelled a few centimetres under the skin. The proximal part is inserted into the

pleural space and the distal valve is connected to a vacuum drainage bottle. The IPC permits regular fluid drainage by a health professional or the patient/carer. Generally well tolerated, they can remain *in situ* for weeks to months.

Criteria for modifying or discontinuing allocated intervention
If a patient, randomised to VAT-PD deteriorates to the point that they are not fit enough to undergo VAT-PD, they will be offered an IPC instead. This decision will be at the discretion of the clinical team managing the patient and will be recorded and reported.

For patients randomised to the IPC arm, the IPC may be removed if there is no significant drainage for 4 weeks and no radiological evidence of significant fluid re-accumulation. All recruited patients will be reported.

Papworth Hospital NHS
NHS Foundation Trust

5.6 PARTICIPANT FOLLOW UP

Follow-up visits at 6 weeks, 3, 6 and 12 months post-randomisation are planned to coincide with standard clinical care visits (Table 1).

Table 1 Schedule of Events

Specific Activity	Undertaken by	Screening	Baseline Rando-misation	Intervention (0-3 weeks post randomisation)	6 weeks +/-1 week	3 month +/-1 week	6 months +/-1 weeks	12 months +/-1 weeks
Identify potential participant	Local MDT	x						
Eligibility check (exclusions)	Local MDT	x						
Approach potential participant to discuss study	Local PI	x						
Take informed consent	Local PI		x					
Baseline clinical data collection, including inclusion and exclusion criteria	Local Research Nurse		x					
Randomisation (web or telephone)	Local Research Nurse		x					
VATS-PD or IPC	Appropriate clinician identified by local PI			x				
VAS Scores for dyspnoea and chest pain	Patient daily for 6 weeks then weekly until 12 months		x	x	x	x	x	x
EQ-5D & EORTC QLQ-C30	Patient B/L, intervention day, 6 weeks, 3, 6, 12 months		x	x	x	x	x	x
Review/reporting of patient AEs/SAEs	Local Research Nurse		x	x	x	x	x	x
Qualitative interviews	Local Research Nurse				x			
Clinical Follow up data	Local Research Nurse				x	x	x	x
Health Service and Resource use data	Local Research Nurse			x	x	x	x	x

Papworth Hospital NHS
NHS Foundation Trust

6 DATA HANDLING AND RECORD KEEPING

The trial will be conducted according to the Good Clinical Practice and Standard Operating Procedures of Papworth Trials Unit Collaborative (PTUC) to ensure the monitoring and safety of trial participants and data validity.

6.1 DATA COLLECTION, MANAGEMENT AND ANALYSIS

A secure, restricted-user, trial-specific database will be developed at PTUC using OpenClinica. Research Nurses will enter the data into the database remotely. Secure data transfer will be used to ensure that patient data submissions are protected and only trial personnel will have access to the files.

Patients will record VAS scores in a booklet The local nurse will initially enter the score onto the database and will then send the booklet to PTUC for a second measurement on a monthly basis to ensure consistency of recording.

Statistical analysis will be carried out under the supervision of Professor Linda Sharples.

Health Service and resource use information will also be collected for treatment following randomisation through the follow-up period to 12 months. We will request data on use of primary care from NHS electronic databases (for those participants who provide consent) and compare this with the quality, quantity and specificity of data provided by patients directly. Data will be stored at Papworth Hospital in accordance with PTUC SOPs. Anonymised records will be analysed for completeness at King's College London

6.2 SCREENING AND RECRUITMENT LOGS

The mesothelioma multidisciplinary team at each centre will keep a screening log of

a) all new cases of MPM and
b) all cases of MPM in which TL is present.
c) all cases considered for MesoTRAP with the outcome of their eligibility status

This will be returned to PTUC on a monthly basis.

Using data from multiple centres this will provide:

a) an estimate of the prevalence of TL in MPM (secondary objective)
b) an estimate of the percentage of eligible patients in participating centres, both of which will inform recruitment rates and sample size calculations.

In recognition that TL sometimes develops during the course of the disease rather than being evident at first presentation, each case of TL will be categorised as either a) having trapped lung present upon first presentation/at initial diagnosis or b) developing trapped lung during the course of their illness and identified during routine follow-up.

6.3 BASELINE AND CLINICAL FOLLOW UP DATA

Baseline data will be collected:

- Demographics (age, sex, ethnicity)
- Height (in metres)
- Weight (in kg)
- Weight loss in previous 6 months
- WHO performance status
- Eastern Cooperative Oncology Group (ECOG) performance status
- Pulmonary function (Forced Expiratory Volume in 1 second, FEV1)
- Smoking history
- Asbestos Exposure: Y/N/Unknown, age at first exposure.
- Comorbidities (Y/N/unknown for diabetes mellitus; renal insufficiency; presence of another respiratory comorbidity or presence of a cardiovascular comorbidity)
- Previous malignancies
- Blood test results (FBC, U&E, LFT)
- Diagnosis (age at diagnosis; symptoms; pathology; cytology; type (epithelioid/non-epithelioid); laterality; histology and staging)
- Quality of life measured using the EQ-5D-5L and EORTC QLQC30
- Visual Analogue Scale scores for dyspnoea and chest pain (see below)

Follow up Data will be collected at intervention, 6 weeks, 3, 6 and 12 months post randomisation:

- Visual Analogue Scale scores for dyspnoea. Scales will be completed daily for the first 6 weeks post-randomisation and then weekly until study completion at 12 months.
- Visual Analogue Scale scores for chest pain. Scales will be completed daily for the first 6 weeks post-randomisation and then weekly until study completion at 12 months.
- Quality of Life patient-reported questionnaires: EQ-5D-5L and EORTC QLQC30.
- Survival status (alive on date of follow up *or* date and cause of death)
- Adverse Events assessed according to GCP guidelines and reported as described below.
- Resource use data as detailed below. Particular attention will be paid to procedures required to control pleural fluid post intervention (e.g. subsequent pleural aspirations/drains, re-do IPC, need for VAT-PP) and access to health services.

6.4 COLLECTION OF VAS DATA TO INFORM POWER CALCULATIONS FOR A DEFINITIVE STUDY.

Assessments of dyspnoea and chest pain will be captured using validated 100mm patient-reported VAS scores in which 0mm represents no dyspnoea or chest pain, and 100mm represents maximum dyspnoea or chest pain (Davies 2012). Patients will be shown how to complete VAS scores in clinic and will subsequently self-report at home by making a mark along the line representing their level of dyspnoea or chest pain. Measurements will be taken at approximately the same time each day (midday) and a patient reminder system will be

developed in order to enhance this process. Scales will be completed daily for the first 6 weeks post-randomisation and then weekly until study completion at 12 months. Completed VAS case report forms will be collected at each patient visit, the CRFs will we sent to PTUC and the data will be measured and entered onto the database.

We aim to understand the patterns of change in VAS scores from randomisation to treatment and to the end of follow-up in order to inform the design of a full definitive study in which the primary (patient-reported) outcome measure will utilise the 100mm VAS. Interpretation of the VAS scores will be aided by recent work defining the minimal important difference (MID) in patients with malignant pleural effusion (Mishra *in press*).

6.5 Quality of Life

We will collect quality of life data at baseline, intervention (EQ-5D-5L and EORTC QLQC30) and at 6 weeks, 3, 6 and 12 months post-randomisation to assess the burden that each intervention puts on patients. We will also assess the number and quality of the returned forms. This is important in a palliative setting where quality of life is often deteriorating and median survival is measured in months. The main tools, EQ-5D-5L and EORTC QLQC30, are very widely used in cancer studies and will be the straightforward to complete.

6.6 Recording and management of Adverse Events

All Unexpected Serious Adverse Events (SAE) occurring between randomisation and the end of follow-up will be recorded in the patient's hospital notes and submitted, within 24 hours of the site becoming aware, to the PTUC using an SAE form on OpenClinica.

All recorded SAEs will be reported to the Sponsor and the Data Monitoring Committee (DMC). If an SAE occurs that is considered to be both unexpected and related to the study protocol (SUSAR), it will be reported within 24 hours of recognition.

Non-serious Adverse Events will be not be recorded or reported for the MesoTRAP trial, unless they form part of the clinical event dataset.

The Sponsor will report any SUSARs to the Research Ethics Committee within 15 days of their knowledge of the event and local PIs will be notified.

Details of Expected Adverse Events are listed in Appendix 1.

6.7 Resource Use Data

For this feasibility study, assessment of health service and resource use data will be limited to:

a) Designing bespoke data collection forms required to collect resource use data at the patient-level for a phase III trial.

b) Comparing the bespoke data collection forms designed to capture resource use associated with the treatments with data collected routinely in centres e.g. for time and resource use during procedures (in theatre or procedure room), length of stay by place (e.g., ICU, ward, procedure room, drug use, complications).

Papworth Hospital NHS
NHS Foundation Trust

c) Requesting information from primary care and hospital trusts to investigate data capture from routine clinical databases for follow-up services (including hospital bed use, potential use of community services, radiotherapy, chemotherapy, hospice care and diagnostic tests). We will compare completeness, specificity, cost, availability and timeliness of the electronic data with that captured on CRFs.

6.8 DATA MONITORING PLANS

The study will be monitored according to the Standard Operating Procedures of Papworth Trials Unit Collaborative (PTUC) as agreed with the Trial Steering and Data Monitoring committees during the study set-up phase.

7 STATISTICS

Statistical analysis will be carried out by under the supervision of Professor Linda Sharples.

Primary analysis

The standard deviation for the VAS dyspnoea scale score to be used in a phase III trial will be estimated as the 70% upper limit of the confidence interval as recommended in Browne (1995). The patterns of change over time will be assessed using descriptive statistics and where appropriate, graphical representations.

Secondary analyses.

i. The standard deviation for the VAS chest pain scale score to be used in a phase III trial will be estimated as the 70% upper limit of the confidence interval as recommended in Browne (1995). The patterns of change over time will be assessed using descriptive statistics and where appropriate, graphical representations.
ii. EQ-5D-5L and EORTC QLQC30 scores will be summarised by treatment group via descriptive statistics in order to examine QoL post-intervention. Scores will also be summarised descriptively and where appropriate, graphically over time in order to assess patterns of change.
iii. The survival rate at 30 days and 12 months post-randomisation will be summarised by treatment arm.
iv. Serious Adverse Events will be recorded from randomisation until the end of the follow up period and will be reported by treatment group via descriptive statistics.
v. The recruitment rate will be estimated as the number of patients with TL due to MPM recruited, divided by the number of respective patients identified as eligible and will be expressed as a rate per centre, per month open for recruitment.
vi. The prevalence of trapped lung in patients with MPM will be estimated as the number of MPM patients with pleural effusion and TL divided by the number of MPM patients within the 18 month study period. This will be multiplied by 100 and reported as a percentage.
vii. The percentage of eligible patients in participating centres will be estimated as the number of eligible patients divided by the number of MPM patients with TL screened in each centre, multiplied by 100.

Papworth Hospital NHS
NHS Foundation Trust

8 HEALTH ECONOMICS

We will investigate the feasibility of alternative data collection mechanisms for health service and resource use data, with a view to setting out a feasible plan for the collection and analysis of cost-effectiveness alongside a future phase III trial. The pilot clinical trial and feasibility study will a) compare local data capture systems within theatres, ICUs and hospital records in centres to check completeness of information prior to finalising the bespoke data collection form for a future trial; b) investigate the data capture processes for follow-up services (including hospital bed use, use of community services, radiotherapy, chemotherapy, hospice care and diagnostic tests) through seeking individual informed consent for access to participants' primary care records and hospital records through trusts and comparing this with self-reported data from patients. c) investigate the EQ-5D-5L (Herdman et al 2011) for data completeness and floor/ceiling effects, with a view to undertaking a future cost-utility analysis.

Papworth Hospital NHS
NHS Foundation Trust

9 Qualitative Assessment Sub Study (QASS)

9.1 QASS Introduction

A QASS will examine patient experience of the interventions and factors influencing patient decisions to participate and accept randomisation or not. This will inform future study design.

After randomisation patients will be invited to participate in the QASS but it will not affect their participation in the main study. We will recruit 5 patients randomised to VAT-PD, 5 randomised to IPC and 5 who declined to participate.

A separate Patient information sheet will be developed and patients will have at least 24 hours to consider participation before an interview is arranged.

Patients who decline randomisation will be asked to consent to be approached about their experience of care or subsequent treatment and reasons for declining to be randomised.

Initially, we will sample purposively to obtain a range of patients in terms of key characteristics that will influence experience (e.g. age, gender, IPC/VAT-PD group).

Areas to be addressed:

- What is the patient experience of the recruitment process?
- What factors influence patient decisions regarding MesoTRAP including participation and randomisation?
- What were the patients preferences regarding interventions
- What is the patient experience of the study interventions?
- What is the impact of participation /intervention (IPC/VAT-PD) e.g. travel to surgical centres if one is not close by, cost of travel, disturbance to daily life/family/work, impact on carers
- What are the implications of the findings for MesoTRAP if it moves to a full study in terms of design, patient information and support?

Interviews will be conducted by trained members of the study team, by telephone or at hospital visits using a topic guide developed with reference to the Trial Steering Group, lay consultation and relevant literature. These will take place about 6-8 weeks post-randomisation (i.e. about 4-6 weeks post intervention for participants) and about 6 weeks post consent discussion for non-participants.

Interviews will take about 45 minutes and will be recorded, transcribed and patient identifiable information removed. Framework analysis methods will be used to study the data and generate key themes (Ritchie 1994).

Papworth Hospital NHS
NHS Foundation Trust

10 Observational Sub-Study

10.1 Observational Sub-Study Introduction

In parallel with the main study an observational sub-study will collect observational data on a cohort of patients who have MPM and trapped lung, but who are either not eligible or decline to participate in the main study. It is expected that a significant proportion of patients with trapped lung will fall into this group. With little existing information on the condition of trapped lung in MPM the aim is to begin to provide high quality data about patients with trapped lung in order to help inform future practice and research.

Patients in the Observational Sub-study will receive the same baseline and follow-up visits as those in the main study, whilst receiving standard clinical care.

10.2 Eligibility

Inclusion Criteria:

1. Pathologically confirmed MPM
2. Trapped lung, defined as a *"clinically significant trapped lung in the opinion of the clinical team"*
3. Age ≥ 18 years
4. Able to provide informed consent

Exclusion Criteria:

1. Lung re-expands fully following pleural fluid drainage i.e. no entrapment

10.3 Recruitment and Follow-up

Patients meeting the eligibility criteria will be informed about the study, provided with a Patient Information Sheet (PIS) and given at least 1 hour to consider participation (there is a separate PIS and Consent Form for this sub-study).

A member of the research team will address any questions and take written informed consent. Informed consent will include permission to access participants' primary care hospital records through trusts as well as self-reported data from patients.

Following provision of consent, the baseline visit will be conducted. Follow-up visits at 6 weeks, 3, 6 and 12 months post-baseline are planned to coincide with standard clinical care visits (Table 2).

Papworth Hospital NHS
NHS Foundation Trust

Patients may also choose to enter the Observational Sub-study but without having to take an active role. In this case they will not be required to complete the VAS scores or quality of life questionnaires and will give permission for their routine clinical data to be collected only.

Table 2 Schedule of Events

Specific Activity	Undertaken by	Screening	Baseline	6 weeks +/-1 week	3 month +/-1 week	6 months +/-1 weeks	12 months +/-1 weeks
Identify potential participant	Local MDT	x					
Eligibility check (exclusions)	Local MDT	x					
Approach potential participant to discuss study	Local PI	x					
Take informed consent	Local PI		x				
Baseline clinical data collection, including inclusion and exclusion criteria	Local Research Nurse		x				
VAS Scores for dyspnoea and chest pain (active participants only)	Patient daily for 6 weeks then weekly until 12 months		x	x	x	x	x
EQ-5D & EORTC QLQ-C30 (active participants only)	Patient B/L, intervention day, 6 weeks, 3, 6, 12 months		x	x	x	x	x
Review/reporting of patient AEs/SAEs	Local Research Nurse		x	x	x	x	x
Qualitative interviews (check eligibility)	Local Research Nurse			x			
Clinical Follow up data	Local Research Nurse			x	x	x	x
Health Service and Resource use data	Local Research Nurse			x	x	x	x

Papworth Hospital NHS
NHS Foundation Trust

11 PROJECT MANAGEMENT

11.1 RESEARCH MANAGEMENT AND GOVERNANCE

The Senior R&D Manager based at Papworth Trials Unit Collaboration (PTUC) will oversee the study.

The Trial Manager(s) will co-ordinate all trial-related activities across the participating sites, monitor progress against the project milestones, ensure full engagement with PPI and manage the finances.

Data management activities will be carried out by PTUC.

The health economic investigation will be carried out at King's College London under the supervision of Professor J Fox-Rushby with whom we have previously collaborated on the MesoVATS study.

11.2 QUALITATIVE SUB STUDY

Professor Angela Tod, University of Sheffield will oversee the QASS. Professor Tod is an experienced qualitative health services researcher who has expertise in research exploring treatment access and decision making in lung cancer and mesothelioma.

11.3 PATIENT AND PUBLIC INVOLVEMENT

Our PPI colleagues will be fully involved with the trial steering group and study write-up.

11.4 STUDY REGISTRATION

The study will be registered with an International Standard Randomised Controlled Trial Number (ISRCTN), and with ClinicalTrials.gov.

11.5 TRIAL MANAGEMENT GROUP (TMG)

A TMG responsible for day-to-day running of the study will meet at least every 2 months by teleconference to discuss recruitment, safety, data management and local site issues.

The TMG will comprise the Chief Investigator, co-applicants, the trial manager, health economist, statistician, qualitative researcher, data manager and representatives from each site.

11.6 TRIAL STEERING COMMITTEE (TSC)

The TSC will meet six monthly (or more frequently if necessary) to monitor and supervise the trial, to ensure it is being conducted according to the protocol and timelines, to review any relevant information from other sources (e.g. other related trials) and to consider recommendations from the DMC.

TSC membership will comprise an independent chair as well as a surgeon, respiratory physician, radiologist, statistician, health economist, qualitative research, trial manager, data manager and an independent patient advocate (PPI).

11.7 Data Monitoring Committee (DMC)

Annual DMC meetings will review progress against the agreed milestones, recruitment and safety. The committee will consist of experienced, independent personnel.

The DMC will meet after the first 15 patients are randomised to review the data for safety. Meetings will be held as necessary should urgent issues arise.

The DMC will develop a charter that describes the framework within which it will operate. The independent members will comprise a statistician (Chair), a surgeon, an oncologist and a respiratory physician.

11.8 CRN Eastern

Our primary linkage will be with Division 1 of the Eastern Clinical Research Network (CRN). The Lead Applicant and Chief Investigator, Dr Rintoul, is part funded by the Eastern CRN.

12 Ethical & Research Governance approvals

12.1 Initial REC and HRA Approval

The protocol and all patient-facing documentation will be submitted to a Research Ethics Committee (REC) and for Health Research Authority (HRA) approval prior to study commencement. HRA Approval is the process for the NHS in England that brings together the assessment of governance and legal compliance with the independent REC opinion provided through the UK research ethics service.

12.2 Site Capability and Capacity

HRA approval replaces the need for local checks of legal compliance and related matters by each participating organisation in England. This allows participating organisations to focus their resources on assessing, arranging and confirming their capacity and capability to deliver the study. The Trial manager will work with the Sponsor to assist local sites with study set up in line with the HRA approval process.

For further information see: http://www.hra.nhs.uk/documents/2015/11/assess-arrange-confirm-clarifications-hra-terminology.pdf

Papworth Hospital NHS
NHS Foundation Trust

12.3 Protocol amendments

Substantial amendments to the protocol and any patient-facing documentation will be submitted to a Research Ethics Committee (REC) and Health Research Authority for approval prior to implementation,

Amendments may only be implemented after a copy of the HRA approval letter has been obtained and local R&D departments have confirmed capacity to accommodate the amendment at that site.

Amendments intended to eliminate an immediate hazard to subjects may be implemented prior to receiving REC approval. However, in this case, approval must be obtained as soon as possible after implementation.

13 Insurance

Centres will be covered by NHS indemnity for negligent harm providing researchers hold a contract of employment with the NHS, including honorary contracts held by academic staff.

14 Publication Policy

The findings of this research will be disseminated in a variety of ways, which we used successfully following completion of the MesoVATS study.

1. The work will be submitted to major national and international clinical and scientific meetings such as a) the International Association for the Study of Lung Cancer (IASLC) annual meeting, b) Bi-annual International Mesothelioma Interest Group (IMIG) meeting, c) British Thoracic Society annual meeting, d) annual NCRI cancer conference, e) annual British Thoracic Oncology Group meeting f) National Lung Cancer Forum for Nurses. This strategy will bring the research to the attention of the majority of clinicians and researchers involved in mesothelioma.

2. We will aim to publish the outputs of the research in an international peer-reviewed journal that is compliant with the policy on open access.

3. We will inform mesothelioma patient/carer support groups of the results including Mesothelioma UK, Clydeside Action on Asbestos, Mick Knighton Mesothelioma Research Fund and the Greater Manchester Asbestos Victims Support Group, a number of whom produce newsletters for their members/supporters.

4. We will inform local clinical multidisciplinary teams (MDTs) via the regional Strategic Clinical Networks and the Academic Health Science Networks.

5. The Trial Steering Committee will agree a formal publication policy for the MesoTRAP study.

Papworth Hospital NHS
NHS Foundation Trust

15 References

http://www.hse.gov.uk/Statistics/causdis/mesothelioma/index.htm (accessed 170115)

Ades A, Lu G, Claxton K. Expected Value of Sample Information Calculations in Medical Decision Modeling Med Decis Making March 2004 24: 207-227, doi:10.1177/0272989X04263162

Allmark P, Boote J. Chambers E. Clarke A. McDonnell A. Thompson A. Tod AM (2009) Ethical issues in the use of in-depth interviews: literature review and discussion. *Research Ethics Review.* Vol 5, No 2, 43-88 10.1177/174701610900500203

Bazerbashi S, Villaquiran J, Awan MY, Unsworth-White MJ, Rahamim J, Marchbank A. Ambulatory intercostal drainage for the management of malignant pleural effusion: a single center experience. Ann Surg Oncol. 2009 Dec;16(12):3482-7. doi: 10.1245/s10434-009-0691-2. Epub 2009 Sep 24

Bottomley A, Gaafar R, Manegold C, Burgers S, Coens C, Legrand C, Vincent M, Giaccone G, Van Meerbeeck J; EORTC Lung-Cancer Group; National Cancer Institute, Canada. Short-term treatment-related symptoms and quality of life: results from an international randomized phase III study of cisplatin with or without raltitrexed in patients with malignant pleural mesothelioma: an EORTC Lung-Cancer Group and National Cancer Institute, Canada, Intergroup Study. J Clin Oncol. 2006 Mar 20;24(9):1435-42. Epub 2006 Jan 30.

Browne R H. On the use of a pilot sample for sample size determination. Statistics in Medicine 1995; 14:1933-1940.

Chee A, Tremblay A The use of tunneled pleural catheters in the treatment of pleural effusions Current Opinions in Pulmonary Medicine 2011;17 (4);237-241

Curran D, Sahmoud T, Therasse P, van Meerbeeck J, Postmus PE, Giaccone G. Prognostic factors in patients with pleural mesothelioma: the European Organization for Research and Treatment of Cancer experience. J Clin Oncol. 1998 Jan;16(1):145-52. Review

Davies HE, Mishra EK, Kahan BC, Wrightson JM, Stanton AE, Guhan A, Davies CW, Grayez J, Harrison R, Prasad A, Crosthwaite N, Lee YC, Davies RJ, Miller RF, Rahman NM. Effect of an indwelling pleural catheter vs chest tube and talc pleurodesis for relieving dyspnea in patients with malignant pleural effusion: the TIME2 randomized controlled trial. JAMA. 2012 Jun 13;307(22):2383-9. doi: 10.1001/jama.2012.5535.

Efthymiou CA, Masudi T, Thorpe JA, Papagiannopoulos K. Malignant pleural effusion in the presence of trapped lung. Five-year experience of PleurX tunnelled catheters. Interact Cardiovasc Thorac Surg. 2009 Dec;9(6):961-4. doi: 10.1510/icvts.2009.211516. Epub 2009 Jul 28.

Herdman, M. et al. “Development and Preliminary Testing of the New Five-Level Version of EQ-5D (EQ-5D-5L).” *Quality of Life Research* 20.10 (2011): 1727–1736.

Papworth Hospital NHS
NHS Foundation Trust

Hodgson JT, McElvenny DM, Darnton AJ, Price MJ, Peto J. The expected burden of mesothelioma mortality in Great Britain from 2002 to 2050. Br J Cancer. 2005 Feb 14;92(3):587-93. Kind P. The EuroQoL instrument: an index of health-related quality of life. In Spilker B, ed. Quality of life and pharmacoeconomics in clinical trials, 2nd edn. Philadelphia, PA: Lippincott-Raven Publishers, 1996:191-201

Kulkarni V, Fernandez-Bussy S, Jantz MA Medical thoracoscopy with simultaneous placement of a PleurX catheter for management of malignant pleural effusion Chest 2009; 136(4). McBrearty A, Cooper D, Bourke SC, Parker SM Audit of medical thoracoscopy at North Tyneside General Hospital Lung Cancer 2012;75(S16)

McCulloch P, Altman DG, Campbell WB, Flum DR, Glasziou P, Marshall JC, Nicholl J, Aronson JK, Barkun JS, Blazeby JM, Boutron IC, Campbell WB, Clavien PA, Cook JA, Ergina PL, Feldman LS, Flum DR, Maddern GJ, Nicholl J, Reeves BC, Seiler CM, Strasberg SM, Meakins JL, Ashby D, Black N, Bunker J, Burton M, Campbell M, Chalkidou K, Chalmers I. et al. No surgical innovation without evaluation: the IDEAL recommendations. Lancet. 2009;374:1105–1112.

Mishra EK, Corcoran J, Hallifax R, Stradling J, Maskell N, Rahman N Defining the minimal important difference for the visual analogue scale assessing dyspnea in patients with malignant pleural effusions. In press 2015

Pennell, C. P., Hirst, A. D., Campbell, W. B., Sood, A., Agha, R. A., Barkun, J. S. T., McCulloch, P. Practical guide to the Idea, Development and Exploration stages of the IDEAL Framework and Recommendations. Br J Surg. 2016, 103:5,1365-2168 Doi:10.1002/bjs.10115

Qureshi RA, Collinson SL, Powell RJ, Froeschle PO, Berrisford RG. Management of malignant pleural effusion associated with trapped lung syndrome. Asian Cardiovasc Thorac Ann. 2008 Apr;16(2):120-3.

Rathinam S, Waller DA. Pleurectomy decortication in the treatment of the "trapped lung" in benign and malignant pleural effusions. Thorac Surg Clin. 2013 Feb;23(1):51-61, vi. doi: 10.1016/j.thorsurg.2012.10.007.

Rintoul RC, Ritchie AJ, Edwards JG, Waller DA, Coonar AS, Bennett M, Lovato E, Hughes V, Fox-Rushby JA, Sharples LD; MesoVATS Collaborators. Efficacy and cost of video-assisted thoracoscopic partial pleurectomy versus talc pleurodesis in patients with malignant pleural mesothelioma (MesoVATS): an openlabel, randomised, controlled trial. Lancet. 2014 Sep 20;384(9948):1118-27. doi: 10.1016/S0140-6736(14)60418-9. Epub 2014 Jun 16.

Ritchie J and Spencer L. (1994) Qualitative data analysis for applied policy research in: Bryman A, Burgess G (eds) Analysing Qualitative Data London. Routeledge pp. 173-194.

Scherpereel A, Astoul P, Baas P, Berghmans T, Clayson H, de Vuyst P, Dienemann H, Galateau-Salle F, Hennequin C, Hillerdal G, Le Péchoux C, Mutti L, Pairon JC, Stahel R, van Houtte P, van Meerbeeck J, Waller D, Weder W; European Respiratory Society/European Society of Thoracic Surgeons Task Force. Guidelines of the European Respiratory Society and the European Society of Thoracic Surgeons for the management of malignant pleural

mesothelioma. Eur Respir J. 2010 Mar;35(3):479-95. doi: 10.1183/09031936.00063109. Epub 2009 Aug 28.

Teare DM, Dimairo M, Shephard N, Hayman A, Whitehead A and Walters SJ. Sample size requirements to estimate key design parameters from external pilot randomised controlled trials: a simulation study. Trials. 2014; 15: 264

Vogelzang NJ, Rusthoven JJ, Symanowski J, Denham C, Kaukel E, Ruffie P, Gatzemeier U, Boyer M, Emri S, Manegold C, Niyikiza C, Paoletti P. Phase III study of pemetrexed in combination with cisplatin versus cisplatin alone in patients with malignant pleural mesothelioma. J Clin Oncol. 2003 Jul 15;21(14):2636-44

16 APPENDIX 1: DEFINITIONS OF ADVERSE EVENTS

16.1 ADVERSE EVENT (AE)

Any untoward medical occurrence or effect in a patient treated on a trial protocol, which does not necessarily have a causal relationship with this treatment.

16.2 SERIOUS ADVERSE EVENT

An adverse event that:

- Results in death
- Is life threatening
- Requires admission to hospital or prolongation of hospitalisation
- Results in persistent or significant disability/incapacity
- Is otherwise medically significant
- Return to theatre or ITU

16.3 EXPECTED MORBIDITY

16.3.1 **Expected morbidity following VATS-PD surgery can include:**

- Pain
- Bleeding
- Infection
- Deep vein thrombosis or pulmonary embolism
- Renal insufficiency
- Myocardial Infarction
- Stroke
- Pleural sepsis (Empyema)
- Atrial Fibrillation

As with all major surgery there is also a risk of death. The risk of in-hospital death with pleurectomy decortication is three per hundred (SCTS 2011).

All in hospital deaths will be reviewed by the Data Monitoring Committee.

16.3.2 **Expected morbidity with an IPC can include:**

- Pleural space infection
- Bleeding from drain
- Infection at insertion site
- Pain at insertion site
- Dislodged/broken drain, blocked drain
