# Supplementary material for: MesoTRAP: a feasibility study that includes a pilot clinical trial comparing video-assisted thoracoscopic partial pleurectomy decortication with indwelling pleural catheter in patients with trapped lung due to malignant pleural mesothelioma designed to address recruitment and randomisation uncertainties and sample size requirements for a phase III trial
Source: BMJ Open Respir Res. 2019 Jan 5;6(1):e000368. doi: 10.1136/bmjresp-2018-000368 (PMC6326291; doi:10.1136/bmjresp-2018-000368)
Supplement: Supplementary data [file bmjresp-2018-000368supp002.pdf]

PATIENT INFORMATION SHEET

Version 3.0, dated 29th May 2018

MesoTRAP: A pilot clinical trial and feasibility study comparing video-assisted thoracoscopic partial pleurectomy/decortication with indwelling pleural catheter in patients with trapped lung due to malignant pleural mesothelioma designed to address recruitment and randomisation uncertainties and sample size requirements for a Phase III trial. IRAS number: XXXXXX

We would like to invite you to take part in our research study. Before you decide, it is important that you understand why the research is being done and what it will involve. ***One of our team will go through the information sheet with you and answer any questions you have.*** Feel free to discuss it with others if you wish. Ask us if there is anything that is not clear or if you want more information. Please take your time to read the following information carefully and decide if you wish to participate.

1. What is the purpose of the study?

Malignant pleural mesothelioma is a cancer, caused by asbestos, which currently affects 2500 people in the UK each year. The main symptom is breathlessness caused by fluid building up in the space between the lung and the chest wall (pleural effusion). Treatment involves draining the fluid to allow the lung to re-expand (pleurodesis). However, sometimes tumour growth over the surface of the lung can prevent it from re-expanding. This ‘trapped’ lung results in fluid re-accumulation and repeated drainage which can lead to discomfort and multiple hospital visits.

One approach to dealing with ‘trapped’ lung in mesothelioma is to insert a thin tube (Indwelling Pleural Catheter – IPC) into the space around the lung. The tube can stay in place for a long time allowing patients to drain off fluid at home.

Another approach is a keyhole surgical operation (video-assisted thoracoscopic partial pleurectomy/decortication – VAT-PD) to remove as much tumour as possible from the lining of the lung to allow it to re-expand.

While both approaches are currently offered in clinical practice, it is not known which of the two is most effective at relieving breathlessness. The only way to find out is to conduct a research trial comparing the two. We plan to do this, but first of all we need to carry out a small pilot study to collect information necessary to help us plan the full study.

We are inviting you to take part in this pilot clinical trial and feasibility study to help us plan a larger research study.

2. Why have I been invited?

You have been invited to take part in our study because you have malignant pleural mesothelioma and pleural effusion with a trapped lung and your doctor thinks you may benefit from either IPC or VAT-PD treatment.

3. Do I have to take part?
No. Your participation in this study is entirely voluntary and you are under no pressure to take part. If you do not wish to take part, the standard of care that you receive will not be affected.

If you do decide to take part, you are still free to withdraw from the study at any time without having to explain why. However, if this were to happen, we would like your permission to continue providing the study researchers with basic information about your progress that would be routinely recorded in your medical records.

4. What will happen to me if I take part?
If you decide to take part you will be asked to sign a Consent Form and will be given a copy of this to keep, together with this Patient Information Sheet. You will have some baseline information taken, tests to measure your lung function, and blood tests taken. You will also be asked to complete a quality of life questionnaire.

A computer programme will then be used to decide randomly (i.e. by chance, like tossing a coin) which one of two groups you will be put into:

- **Group 1: Video-assisted thoracoscopic partial pleurectomy/decortication (VAT-PD)**

or

- **Group 2: Indwelling pleural catheter (IPC)**

Neither you nor your doctor will be able to choose which group you are put into. This is so that we can make a fair and unbiased comparison, with groups well-balanced for relevant patient characteristics such as state of health.

Group 1. Video-assisted thoracoscopic partial pleurectomy/decortication (VAT-PD)
If you are allocated to this group, you will be given a date for your operation. The operation may take place at the centre that you have been attending for your mesothelioma, if it has specialist VAT-PD surgical facilities on site. However, it may be that you are referred to another centre that specialises in VAT-PD surgery at a different location. If this is the case, it will mean you will have to attend the other centre for your operation. (This would be the case even if you were not taking part in this research and were referred by your doctor for VAT-PD surgery as part of your standard care).

What does the VAT-PD procedure involve?
VAT-PD is a type of "keyhole surgery" performed under general anaesthesia by a highly-specialised thoracic surgeon, using a telescope and instruments put inside the chest. Through small incisions, or keyholes made between the ribs, the thoracic surgeon removes the hard rind of the tumour over the surface of the lung, thereby allowing the 'trapped' lung to fully expand again. Simultaneous removal of mesothelioma from the outer pleural membrane allows pleurodesis to occur. At the end of the operation, one or more chest drain are placed in the pleural space, and these are left in, to drain off any further collections of fluid or unwanted air.

Group 2: Indwelling pleural catheter (IPC)
If you are allocated to this group, you will be scheduled to have an IPC inserted as a day-case at the centre you have been attending for your mesothelioma.

What does the IPC procedure involve?
An IPC is a soft silicone catheter with a one-way valve at the end, which is inserted a few centimetres under the skin under local anaesthesia. The inside end of the catheter is inserted into the pleural space and the outside end is connected to a vacuum drainage bottle. The IPC permits regular fluid drainage by a health professional or the patient/carer. Generally well-tolerated, they can drain fluid for weeks to months until fluid production ceases and the IPC can be removed.

In the event that you develop symptoms due to a pleural effusion while awaiting either procedure to be performed, fluid will be drained as per standard care but you will go on to receive the procedure of the group you were assigned to. While IPC insertion is likely to be performed within a few days, patients may wait 2-3 weeks for a VAT-PD procedure. If you are assigned to the VAT-PD group and your condition changes such that you become no longer suitable for this procedure, you will be offered an IPC instead. This decision will be made by the doctors who are caring for you.

5. What will I have to do?

From the point at which you are allocated to your treatment group, we ask that you record how you feel in terms of breathlessness and chest pain in a diary. A study doctor or nurse will show you how to do this. You will be asked to record these scores at home, every day for the first 6 weeks following your treatment allocation, and then weekly until the study is completed at 12 months..

You will be asked about your quality of life and how your health has been at 6 weeks, 3 months, 6 months, and 12 months after your procedure. Each session will take about 60 minutes of your time and will take place at the centre you have been receiving treatment at (or, where this is not feasible, over the telephone). All efforts will be made to coincide study visits with any routine visits you would be attending anyway as part of your standard care.

"Patient Experience" Interview Sub-Study: A few patients will be invited to take part in the "Patient Experience" Interview Sub-Study. If you are invited to take part in this additional study, you will have the opportunity to ask questions about the study and will be given a separate Patient Information Sheet to read and a separate Consent Form to sign. You are under no obligation to take part in the "Patient Experience" interview.

6. What are the alternatives for diagnosis and treatment?

If you do not wish to take part in this research, your doctor will offer you the most appropriate care.

7. What are the possible disadvantages and risks of taking part?

At present, there is no agreement on what is considered the 'routine care' and both VAT-PD surgery and IPC insertion are used in trapped lung. Both procedures have pros and cons.

The advantage of VAT-PD surgery is that trapped lung and pleurodesis are dealt with in one procedure, but disadvantages include having to stay in hospital for up to 7 days and approximately 1 in 5 patients will have a complication requiring a longer stay or further treatment.

Because VAT-PD is a highly specialised type of surgery performed at fewer centres in the UK, it is possible that you will have to receive this surgery at a centre other than the one you are used to going to for your mesothelioma. Your doctor and care team looking after you will organise this.

The advantages of receiving an IPC are that these are generally well tolerated and can drain fluid for weeks to months until fluid production ceases and the IPC can be removed. However, complications such as infection in the space around the lung (13% of cases), tube blockage (20% of cases) and displacement can occur, requiring removal or replacement. For some patients, the presence of the catheter acts as a constant reminder of the underlying disease. Placement of an IPC is also dependent upon availability of a community-based health care professional or patient/carer to drain fluid 2-3 times weekly.

From the point at which you are allocated to your treatment group (whether it is VAT-PD surgery or IPC insertion), you will need to record regular information about your levels of breathless and chest pain as well as have study follow up sessions with a study doctor or nurse at 6 weeks, 3 months, 6 months, and 12 months. These follow up sessions will take about 60 minutes of your time and will take place at the centre you have been receiving treatment at (or, where this is not feasible, over the telephone). All efforts will be made to co-incide study visits with any routine visits you would be attending anyway as part of your standard care.

8. What are the possible benefits of taking part?
You may or may not benefit from taking part in this research. You will receive treatment for your trapped lung using one technique or the other and be closely monitored following your treatment.

The information we obtain from this study will assist us with your care and will help us develop the full study which is aimed at improving the care of future patients.

9. What happens when the research study stops?
When the research study stops, your clinical care will continue as normal. If you have an IPC in place when the study ends, you will continue to have this managed in both hospital and community settings as required.

10. What if relevant new information becomes available?
Sometimes we get new information about the treatment being studied. If this happens, your research doctor will tell you and discuss whether you should continue in the study. If you decide not to carry on, your research doctor will make arrangements for your care to continue.

11. What will happen if I don’t want to carry on with the study?
You are free to withdraw from the study at any time without having to explain why. However, if you decide to withdraw, we would like your permission to continue providing the study researchers with some basic information about your progress that would be recorded routinely in your medical records.

12. What if there is a problem?
If you are concerned about any aspect of this study you should ask to speak to one of the researchers (contact details below) who will do their best to answer your questions. If you remain unhappy and wish to complain formally, you can do this through the NHS Complaints Procedure. Details can be obtained from the Patient Advice and Liaison Service (PALS) (contact details below*)*.

If something goes wrong and you are harmed during the study due to someone’s negligence then you may have grounds for a legal action for compensation against the hospital involved,

but you may have to pay your legal costs. The normal NHS complaints mechanisms will still be available to you.

NHS hospitals are unable to agree in advance to pay compensation for non-negligent harm (situations where no one can be blamed for what happened). However, NHS Trusts are able to consider offering an ex-gratia payment in the case of a claim.

13. Will my taking part in this study be kept confidential?

All information that is collected about you during the course of this study will be kept strictly confidential according to the General Data Protection Regulation (GDPR).

Royal Papworth Hospital is the sponsor for this study based in the United Kingdom. We will be using information from you and your medical records in order to undertake this study and will act as the data controller for this study. This means that we are responsible for looking after your information and using it properly. Royal Papworth Hospital will keep identifiable information about you for 15 years after the study has finished.

Your rights to access, change or move your information are limited, as we need to manage your information in specific ways in order for the research to be reliable and accurate. If you withdraw from the study, we will keep the information about you that we have already obtained. To safeguard your rights, we will use the minimum personally-identifiable information possible.

Information on paper will be kept in locked filing cabinets and where possible behind security coded, locked doors. Electronic information will be kept on computers that are protected by passwords.

The electronic data we store for this study will be kept on a secure database at Royal Papworth Hospital. You will be allocated a study number, which will be used along with your date of birth and initials to identify you within the database. Your consent form may be sent (usually using standard Royal Mail post but in some cases by fax or email) from your local hospital to Royal Papworth Hospital.

If you agree, your X-ray and CT scan images will be transferred to Royal Papworth Hospital using the Secure Image Exchange Portal system that is routinely used to transfer images between hospitals. These images may contain your name, date of birth, NHS and Local Hospital number, but this information will only be shared with the study team.

Representatives from regulatory authorities may need to look at your medical records and the data collected in the study to check that the study was carried out correctly. All will have a duty of confidentiality to you.

14. What will happen to the results of the research study?
Upon completion of the study, we will let you know the results. It is our intention to publish the results in relevant medical journals as soon as there is enough information to be sure the results are reliable. You will not be identified in any report or publication.

15. Who is organising and funding the research?
The study is being organised by a team of doctors and other experts who look after patients with mesothelioma. It is being led and managed by Royal Papworth Hospital NHS Foundation Trust. It has been funded by a Research for Patient Benefit (RfPB) grant from

the National Institute for Health Research (PB-PG-1014-35050). The lead doctor (Chief Investigator) is Dr Robert Rintoul, Consultant Chest Physician, at Royal Papworth Hospital NHS Foundation Trust. Your doctor will not receive any personal financial payment for including you in this research.

16. Who has reviewed the study?
This study has been reviewed and approved by the National Research Ethics Service Committee (East of England – Cambridge Central); the NHS Health Research Authority and the Research & Development Office at your hospital.

17. Further information and contact details
For further information, you can speak to one of the study team:

Your study doctor is:

Contact phone numbers:

Your research nurse is:

Contact phone number:

Alternatively, you can speak to an independent contact:
Patient Advice and Liaison Service (PALS)

Contact details:

Thank you for considering taking part in this study.

If you decide to participate you will be asked to sign a consent form and will be given a copy of this information sheet and the consent form to keep.

[INSERT HOSPITAL HEADER HERE]

Site Code:
Study Number: P02128
Patient Identification Number:

CONSENT FORM

Version 3.0 29th May 2018

Study title: MesoTRAP feasibility study IRAS number: 198596

Local Investigator:

Please initial box

1. I confirm that I have read and understood the information sheet dated 29th May 2018 (version 3.0) for the above study. I have had the opportunity to consider the information, ask questions and have had these answered satisfactorily. ☐

2. I understand that my participation is voluntary and that I am free to withdraw at any time without giving any reason, without my medical care or legal rights being affected. ☐

3. I understand that relevant sections of my medical notes and data collected during the study may be looked at by responsible individuals from the research team, from regulatory authorities or from the NHS Trust, where it is relevant to my taking part in this research. I give permission for these individuals to have access to my records. ☐

4. I agree to my GP being informed of my participation in the study. ☐

5. If I withdraw from the study, I consent to my doctor providing authorised researchers with basic clinical information that would routinely be collected and written in my medical records. ☐

6. I understand that I may be approached later to take part in a "Patient Experience" Interview Sub-Study. I understand that I am under no obligation to take part in the interview if I do not want to at the time. ☐

7. I agree for my X-ray and CT scan images to be transferred to Royal Papworth Hospital using the Secure IEP system. These images may contain my name, date of birth, NHS and Local Hospital number, but this information will only be shared with the study team. ☐

8. I agree to take part in the above study. ☐

Name of Patient (PRINT)	Date	Signature
Name of person taking consent (PRINT)	Date	Signature

When completed: 1 for participant; 1 for site file; 1 (original) to be kept in medical notes.
