# Supplementary material for: MesoTRAP: a feasibility study that includes a pilot clinical trial comparing video-assisted thoracoscopic partial pleurectomy decortication with indwelling pleural catheter in patients with trapped lung due to malignant pleural mesothelioma designed to address recruitment and randomisation uncertainties and sample size requirements for a phase III trial
Source: BMJ Open Respir Res. 2019 Jan 5;6(1):e000368. doi: 10.1136/bmjresp-2018-000368 (PMC6326291; doi:10.1136/bmjresp-2018-000368)
Supplement: Supplementary data [file bmjresp-2018-000368supp003.pdf]

List of MesoTRAP Sites (August 2018)

Site	Principal Investigator
Barts Health NHS Trust	Mr Kelvin Lau
Blackpool Teaching Hospitals NHS Foundation Trust	Mr M Nidal Bittar
Cambridge University Hospitals	Dr Jurgen Herre
Derby Teaching Hospitals NHS Foundation Trust	Dr Paul Beckett
Golden Jubilee National Hospital	Mr Alan Kirk
Queen Elizabeth University Hospital, Glasgow	Dr Kevin Blyth
University Hospitals of Leicester NHS Trust	Mr Apostolos Nakas
Norfolk and Norwich University Hospitals	Dr Eleanor Mishra
University Hospitals of North Midlands NHS Trust	Dr Shahul Khan
Nottingham University Hospitals NHS Trust	Dr Helen Roberts
Oxford University Hospitals NHS Foundation Trust	Dr Dionisios Stavroulias
The Pennine Acute Hospitals NHS Trust	Dr Louise Brown
Lancashire Teaching Hospitals NHS Foundation Trust	Dr Mohammed Munawar
Manchester University NHS Foundation Trust	Dr Matthew Evison
North Bristol NHS Trust	Dr Nick Maskell
Royal Papworth Hospital NHS Foundation Trust	Dr Robert Rintoul
North West Anglia NHS Foundation Trust	Dr Pasupathy Sivasothy
Sheffield Teaching Hospitals NHS Trust	Mr John Edwards
