# Supplementary material for: MesoTRAP: a feasibility study that includes a pilot clinical trial comparing video-assisted thoracoscopic partial pleurectomy decortication with indwelling pleural catheter in patients with trapped lung due to malignant pleural mesothelioma designed to address recruitment and randomisation uncertainties and sample size requirements for a phase III trial
Source: BMJ Open Respir Res. 2019 Jan 5;6(1):e000368. doi: 10.1136/bmjresp-2018-000368 (PMC6326291; doi:10.1136/bmjresp-2018-000368)
Supplement: Supplementary data [file bmjresp-2018-000368supp004.pdf]

PATIENT INFORMATION SHEET

Version 1.0, dated 15th May 2018

MesoTRAP Observational Sub-Study

An observational study of patients with trapped lung due to malignant pleural mesothelioma. IRAS number: 198596

We would like to invite you to take part in our research study. Before you decide, it is important that you understand why the research is being done and what it will involve. ***One of our team will go through the information sheet with you and answer any questions you have.*** Feel free to discuss it with others if you wish. Ask us if there is anything that is not clear or if you want more information. Please take your time to read the following information carefully and decide if you wish to participate.

1. What is the purpose of the study?
Malignant pleural mesothelioma is a cancer, caused by asbestos, which currently affects 2500 people in the UK each year. The main symptom is breathlessness caused by fluid building up in the space between the lung and the chest wall (pleural effusion). Treatment involves draining the fluid to allow the lung to re-expand (pleurodesis). However, sometimes tumour growth over the surface of the lung can prevent it from re-expanding. This 'trapped' lung results in fluid re-accumulation and repeated drainage which can lead to discomfort and multiple hospital visits.

Currently very little is known about patients with trapped lung and mesothelioma. We are conducting a study to collect observational data on patients with trapped lung and mesothelioma in order to assess the following:

- What kind of tests and treatment you need
- How many visits to healthcare professionals you need
- How your quality of life is affected
- How you feel in terms of chest pain
- How you feel in terms of breathlessness

This sub-study forms part of the larger MesoTRAP Trial, which compares two types of treatment for trapped lung. You may already have declined to take part in the MesoTRAP Trial OR your doctor does not think you are suitable for the main trial but thinks you may be suitable for this Observational Sub-Study.

2. Why have I been invited?
You have been invited to take part in our study because you have malignant pleural mesothelioma with a trapped lung.

3. Do I have to take part?
No. Your participation in this study is entirely voluntary and you are under no pressure to take part. If you do not wish to take part, the standard of care that you receive will not be affected.

If you do decide to take part, you are still free to withdraw from the study at any time without having to explain why

4. What will happen to me if I take part?
If you decide to take part you will be asked to sign a Consent Form and will be given a copy of this to keep, together with this Patient Information Sheet. You will have some baseline information recorded such as height, weight and medical history. We will also collect the results of your routine lung function and blood tests. You will also be asked to complete a quality of life questionnaire.

5. What will I have to do?
From the day on which you give your consent to enter the study, we ask that you record how you feel in terms of breathlessness and chest pain in a diary. A study doctor or nurse will show you how to do this. You will be asked to record these scores at home, every day for the first 6 weeks, and then weekly until the study is completed at 12 months.

You will be asked about your quality of life and how your health has been at 6 weeks, 3 months, 6 months, and 12 months after your baseline information is recorded. Each session will take about 60 minutes of your time and will take place at the centre where you have been receiving treatment (or, where this is not feasible, over the telephone). All efforts will be made to combine any study visits with routine visits you would be attending anyway as part of your standard care.

If you do not want to take an active part in this study you can still give your permission for the study team to collect routine data from your notes. In this case you will still be enrolled in the study but will not have to complete the quality of life questionnaires or chest pain and breathlessness scores.

6. What are the possible disadvantages and risks of taking part?

This is an observational study and your clinical care will not be altered by taking part. Therefore there should be no additional risk to you. Every effort will be made to ensure that your study visits coincide with your routine clinical visits to minimise any inconvenience to you.

7. What are the possible benefits of taking part?
The study will not alter your treatment in any way. There is no payment for taking part in the study. We hope that the information we gain from this study will help to improve the care of future patients.

8. What will happen if I don't want to carry on with the study?
You are free to withdraw from the study at any time without having to explain why. However, if you decide to withdraw, we would like your permission to continue providing the study researchers with some basic information about your progress that would be recorded routinely in your medical records.

9. What if there is a problem?
If you are concerned about any aspect of this study you should ask to speak to one of the researchers (contact details below) who will do their best to answer your questions. If you remain unhappy and wish to complain formally, you can do this through the NHS Complaints Procedure. Details can be obtained from the Patient Advice and Liaison Service (PALS) (contact details below*).*

If something goes wrong and you are harmed during the study due to someone's negligence then you may have grounds for a legal action for compensation against the hospital involved, but you may have to pay your legal costs. The normal NHS complaints mechanisms will still be available to you.

NHS hospitals are unable to agree in advance to pay compensation for non-negligent harm (situations where no one can be blamed for what happened). However, NHS Trusts are able to consider offering an ex-gratia payment in the case of a claim.

10. How will my information be used?

All information that is collected about you during the course of this study will be kept strictly confidential according to the General Data Protection Regulation (GDPR).

Royal Papworth Hospital is the sponsor for this study based in the United Kingdom. We will be using information from you and/or your medical records in order to undertake this study and will act as the data controller for this study. This means that we are responsible for looking after your information and using it properly. Royal Papworth Hospital will keep identifiable information about you for 15 years after the study has finished.
Your rights to access, change or move your information are limited, as we need to manage your information in specific ways in order for the research to be reliable and accurate. If you withdraw from the study, we will keep the information about you that we have already obtained. To safeguard your rights, we will use the minimum personally-identifiable information possible.
You can find out more about how we use your information at papworth.mesotraptrial@nhs.net.

Information on paper will be kept in locked filing cabinets and where possible behind security coded, locked doors. Electronic information will be kept on computers that are protected by passwords.

The electronic data we store for this study will be kept on a secure database at Royal Papworth Hospital You will be allocated a study number, which will be used along with your date of birth and initials to identify you within the database. Your consent form may be sent (usually using standard Royal Mail post but in some cases by fax or email) from your local hospital to Papworth Hospital

If you agree, your X-ray and CT scan images will be transferred to Royal Papworth Hospital using the Secure Image Exchange Portal system that is routinely used to transfer images

between hospitals. These images may contain your name, date of birth, NHS and Local Hospital number, but this information will only be shared with the study team.

Representatives from regulatory authorities may need to look at your medical records and the data collected in the study to check that the study was carried out correctly. All will have a duty of confidentiality to you.

11. What will happen to the results of the research study?
Upon completion of the study, we will let you know the results. It is our intention to publish the results in relevant medical journals as soon as there is enough information to be sure the results are reliable. You will not be identified in any report or publication.

12. Who is organising and funding the research?
The study is being organised by a team of doctors and other experts who look after patients with mesothelioma. It is being led and managed by Royal Papworth Hospital NHS Foundation Trust. It has been funded by a Research for Patient Benefit (RfPB) grant from the National Institute for Health Research (PB-PG-1014-35050). The lead doctor (Chief Investigator) is Dr Robert Rintoul, Consultant Chest Physician, at Royal Papworth Hospital NHS Foundation Trust. Your doctor will not receive any personal financial payment for including you in this research.

13. Who has reviewed the study?
This study has been reviewed and approved by the National Research Ethics Service Committee (East of England – Cambridge Central); the NHS Health Research Authority and the Research & Development Office at your hospital.

14. Further information and contact details

For further information, you can speak to one of the study team:

Your study doctor is:

Contact phone numbers:

Your research nurse is:

Contact phone number:

Alternatively, you can speak to an independent contact:
Patient Advice and Liaison Service (PALS)

Contact details:

Thank you for considering taking part in this study.

If you decide to participate you will be asked to sign a consent form and will be given a copy of this information sheet and the consent form to keep.

[INSERT HOSPITAL HEADER HERE]

Site Code:
Study Number: P02128
Patient Identification Number:

CONSENT FORM

Version 1.0 15th May 2018

Study title: MesoTRAP Observational Sub-Study IRAS number: 198596
Local Investigator:

Please initial box

1. I confirm that I have read and understood the information sheet Version 1.0 dated 15th May 2018 for the above study. I have had the opportunity to consider the information, ask questions and have had these answered satisfactorily. ☐

2. I understand that my participation is voluntary and that I am free to withdraw at any time without giving any reason, without my medical care or legal rights being affected. ☐

3. I understand that relevant sections of my medical notes and data collected during the study may be looked at by responsible individuals from the research team, from regulatory authorities or from the NHS Trust, where it is relevant to my taking part in this research. I give permission for these individuals to have access to my records. ☐

4. I agree to my GP being informed of my participation in the study. ☐

5. If I withdraw from the study, I consent to my doctor providing authorised researchers with basic clinical information that would routinely be collected and written in my medical records. ☐

6. I agree for my X-ray and CT scan images to be transferred to Royal Papworth Hospital using the Secure IEP system. These images may contain my name, date of birth, NHS and Local Hospital number, but this information will only be shared with the study team. ☐

Choose 7 or 8 and delete the incorrect statement.

7. I agree to take part in the above study (including completing the patient diaries and questionnaires) ☐

8. I do not want to take part in the full study but agree to my routine data being collected. ☐

Name of Patient (PRINT)	Date	Signature
Name of person taking consent (PRINT)	Date	Signature

When completed: 1 for participant; 1 for site file; 1 (original) to be kept in medical notes.
