# Supplementary material for: MesoTRAP: a feasibility study that includes a pilot clinical trial comparing video-assisted thoracoscopic partial pleurectomy decortication with indwelling pleural catheter in patients with trapped lung due to malignant pleural mesothelioma designed to address recruitment and randomisation uncertainties and sample size requirements for a phase III trial
Source: BMJ Open Respir Res. 2019 Jan 5;6(1):e000368. doi: 10.1136/bmjresp-2018-000368 (PMC6326291; doi:10.1136/bmjresp-2018-000368)
Supplement: Supplementary data [file bmjresp-2018-000368supp005.pdf]

PATIENT INFORMATION SHEET

Version 2.0 Dated 17th October 2016

MesoTRAP "Patient Experience" Interview Sub-Study

As part of the MesoTRAP study we are conducting a "Patient Experience" Interview Sub-Study. You are being invited to take part in this. Before you decide, it is important that you understand why the research is being done and what it will involve. ***One of our team will go through the information sheet with you and answer any questions you have.*** Feel free to discuss it with others if you wish. Ask us if there is anything that is not clear or if you want more information. Please take your time to read the following information carefully and decide if you wish to participate.

1. What is the purpose of the patient experience interview sub-study?
This patient experience aims to find out:

- the best way of presenting information to patients about the MesoTRAP study and treatments (VAT-PD surgery and IPC)
- the best way to support people with malignant pleural mesothelioma and trapped lung and who are having treatment
- participating patients' experience of taking part in the MesoTRAP trial and their treatment (VAT-PD surgery or IPC)
- the reasons why eligible patients choose to enter or not enter into a study

3. Why have I been invited?
You have been invited to take part in this Patient Experience Sub-Study because:

- you are currently taking part in the MesoTRAP trial and have been randomised to and received either VAT-PD surgery or IPC

  OR

- you declined to take part in the MesoTRAP trial

4. Do I have to take part?
No. It is up to you whether or not you want to take part. Deciding not to take part will not affect the standard of care you receive or your ability to stay in the main MesoTRAP trial. If you decide to take part in this Patient Experience Sub-Study, you will be asked to sign a second consent form stating this.

5. What will happen if I do decide to take part?

If you do agree to take part in the Patient Experience Sub-Study you will be given copies of your consent form to keep, together with this information sheet. If you agree to join the study you are still free to withdraw at any time without giving a reason. If you withdraw at any time this will not affect the standard of care you receive.

6. What will the interview involve?

The interview will take at a place that is convenient to you (e.g., routine clinic visit or at home over the telephone). It will be conducted by trained members of the study team, at about 6-8 weeks after your VAT-PD or IPC treatment (or at about 6 weeks after being approached about the MesoTRAP study, if you are not participating in the main MesoTRAP trial). The interview will last about 45 minutes. Whilst you are talking, the interviewer will make a few notes and the conversation will be recorded on a small recorder. You will be able to ask the interviewer to pause the recording at any time during the interview. You are also free to stop the interview should you wish to do so. At the end of the interview, the interviewer will check once again that you are happy for the conversation to be included in the study.

7. Will my details be kept confidential?

The interviews will be audio recorded and transcribed word for word. All information that could potentially identify you (e.g. your name or address) will be removed so that the transcript is fully anonymised. Both the recording and the transcription will be stored in a safe place for up to five years after the end of the study and then the recording and the written information will be destroyed. All information, which is collected about you during the time of the study, will be kept strictly confidential.

We hope to publish the results of the study in a medical journal, including some anonymised quotations from people who have taken part in the study. If you do not wish for your actual quotations to be used you do not have to agree to this – you will need to tick a separate box on the consent form to agree the use of direct quotes in future publications or presentations.

8. Will I benefit from taking part in this study?

The study will not alter your treatment in any way. There is no payment for taking part in the study. However, we hope that the results of this study will help us to improve the care and support we give to people in future studies.

9. What will happen to the results of the research study?

The results will be published in medical journals as soon as there is enough information to be sure the results are reliable. You will not be identified in any report or publication. The results will help to decide how to treat patients with trapped lung due to malignant pleural mesothelioma in the future.

You will not be identified personally in any presentation or report of this research.

At the end of the study you are welcome to ask the study team for details of any publication(s) relating to the study.

10. Will my taking part in this study be kept confidential?

The collection and submission of all information gathered from this study will be done with adherence to the regulations governing clinical research. If you consent to take part in the

research, all information collected from you during the course of the study will be kept strictly confidential.

If, during the course of the interview, a patient reveals a problem or concern (e.g., deterioration in their condition), the interviewer will let the relevant study team or clinical staff know. This will be done following discussion with the participant.

11. Who is organising and funding the study?
This study is being organised by a team of doctors and other experts who look after patients with trapped lung due to mesothelioma. The Patient Experience Sub-Study is being co-ordinated and run by a research team led by Professor Angela Tod (University of Manchester), in collaboration with the lead MesoTRAP doctor (Chief Investigator), Dr Robert Rintoul, Consultant Chest Physician, at Papworth Hospital NHS Foundation Trust. The study is being led and managed by Papworth Hospital NHS Foundation Trust in collaboration with Leeds Clinical Trial Research Unit. It has been funded by a Research for Patient Benefit (RfPB) grant from the National Institute for Health Research (PB-PG-1014-35050).

12. What if something goes wrong?
If you are concerned about any aspect of this study you should ask to speak to one of the researchers (contact details below) who will do their best to answer your questions. If you remain unhappy and wish to complain formally, you can do this through the NHS Complaints Procedure. Details can be obtained from the Patient Advice and Liaison Service (PALS) (contact details below*).*

If something goes wrong and you are harmed during the study due to someone's negligence then you may have grounds for a legal action for compensation against the hospital involved, but you may have to pay your legal costs. The normal NHS complaints mechanisms will still be available to you.

NHS hospitals are unable to agree in advance to pay compensation for non-negligent harm (situations where no one can be blamed for what happened). However, NHS Trusts are able to consider offering an ex-gratia payment in the case of a claim.

13. Who has reviewed this study?
This study has been reviewed and approved by the National Research Ethics Service Committee (East of England – Cambridge Central) and also by the Research & Development Office at your hospital.

14. Further information and contact details
For further information, you can speak to one of the study team:

Your study doctor is:

Contact phone numbers:

The interview sub-study researcher is:

Contact details:

Alternatively, you can speak to an independent contact:
Patient Advice and Liaison Service (PALS)

Contact details:

Thank you for considering taking part in this study.

If you decide to participate you will be asked to sign a consent form and will be given a copy of this information sheet and the consent form to keep.

[INSERT HOSPITAL HEADER HERE]

Centre Number:
Study Number:
Patient Identification Number:

CONSENT FORM

Version 2.0 Dated 17th October 2016

MesoTRAP "Patient Experience" Interview Sub-Study

Local Investigator:

Please initial box

1. I confirm that I have read and understood the "Patient Experience Interview Sub-Study" information sheet dated 17th October 2016 (version 2.0) for the above study. I have had the opportunity to consider the information, ask questions and have had these answered satisfactorily. ☐

2. I understand that my participation is voluntary and that I am free to withdraw at any time without giving any reason, without my medical care or legal rights being affected. ☐

3. I consent to the audio taping of my interview. ☐

4 I consent to the storage of personal information for the purposes of this study. This may include paper or electronic information. I understand that any information that could identify me will be kept strictly confidential and that no personal information will be included in the study report or other publication. ☐

5. I agree to take part in the above study. ☐

6. I give permission for some of my actual quotations to be used anonymously in any future presentations and publications (optional)

YES/NO

Name of Patient (PRINT)	Date	Signature
Name of person taking consent (PRINT)	Date	Signature

When completed: 1 for participant; 1 for site file; 1 (original) to be kept in medical notes.
